# Using community-based reporting of vital events to monitor child mortality: Lessons from rural Ghana

**DOI:** 10.1371/journal.pone.0192034

**Published:** 2018-01-30

**Authors:** Stephane Helleringer, Daniel Arhinful, Benjamin Abuaku, Michael Humes, Emily Wilson, Andrew Marsh, Adrienne Clermont, Robert E. Black, Jennifer Bryce, Agbessi Amouzou

**Affiliations:** 1 Department of Population, Family, and Reproductive Health, Johns Hopkins Bloomberg School of Public Health, Baltimore, MD, United States of America; 2 Noguchi Memorial Institute for Medical Research, University of Ghana, Legon, Ghana; 3 Institute for International Programs, Johns Hopkins Bloomberg School of Public Health, Baltimore, MD, United States of America; University of Sussex, UNITED KINGDOM

## Abstract

**Background:**

Reducing neonatal and child mortality is a key component of the health-related sustainable development goal (SDG), but most low and middle income countries lack data to monitor child mortality on an annual basis. We tested a mortality monitoring system based on the continuous recording of pregnancies, births and deaths by trained community-based volunteers (CBV).

**Methods and findings:**

This project was implemented in 96 clusters located in three districts of the Northern Region of Ghana. Community-based volunteers (CBVs) were selected from these clusters and were trained in recording all pregnancies, births, and deaths among children under 5 in their catchment areas. Data collection lasted from January 2012 through September 2013. All CBVs transmitted tallies of recorded births and deaths to the Ghana Birth and deaths registry each month, except in one of the study districts (approximately 80% reporting). Some events were reported only several months after they had occurred. We assessed the completeness and accuracy of CBV data by comparing them to retrospective full pregnancy histories (FPH) collected during a census of the same clusters conducted in October-December 2013. We conducted all analyses separately by district, as well as for the combined sample of all districts. During the 21-month implementation period, the CBVs reported a total of 2,819 births and 137 under-five deaths. Among the latter, there were 84 infant deaths (55 neonatal deaths and 29 post-neonatal deaths). Comparison of the CBV data with FPH data suggested that CBVs significantly under-estimated child mortality: the estimated under-5 mortality rate according to CBV data was only 2/3 of the rate estimated from FPH data (95% Confidence Interval for the ratio of the two rates = 51.7 to 81.4). The discrepancies between the CBV and FPH estimates of infant and neonatal mortality were more limited, but varied significantly across districts.

**Conclusions:**

In northern Ghana, a community-based data collection systems relying on volunteers did not yield accurate estimates of child mortality rates. Additional implementation research is needed to improve the timeliness, completeness and accuracy of such systems. Enhancing pregnancy monitoring, in particular, may be an essential step to improve the measurement of neonatal mortality.

## Introduction

Countries worldwide must have accurate data on births and deaths to track progress towards the health-related Sustainable Development Goal (SDG) on an annual basis. Unfortunately, a large number of low- and middle-income countries (LMICs) do not have adequate national civil registration and vital statistics (CRVS) systems to collect such data [1, 2]. Despite recent increases in the coverage of birth registration in a number of LMICs, deaths are seldom registered, particularly among children. Mortality rates thus cannot be estimated reliably from CRVS data in most LMICs [3–6].

Instead, the main sources of mortality estimates in LMICs with limited CRVS systems are a) the health management information system (HMIS) and b) retrospective interviews with survivors, most commonly conducted during household surveys such as the Demographic and Health Surveys (DHS) and Multiple Indicator Cluster Surveys (MICS). The HMIS, however, is highly selective: it only takes into account deaths that occur in health facilities. Retrospective interviews, on the other hand, yield representative estimates of mortality rates for countries or sub-national regions when they are collected during representative surveys. They do so by collecting full pregnancy histories (FPH) or full birth histories (FBH), i.e., a series of questions during which women aged 15–49 years old report on all pregnancies or all births they ever had and the associated dates of birth outcomes and date or age at death of each child who died. [7]. FPH and FBH suffer from potential sample selection biases, because the children whose mother died before the survey cannot be included in a FBH or FPH. In particular, if the survival of the mother and the survival of the child are correlated, then FPH or FBH may under-estimate mortality [8]. FPH and FBH are also subject to reporting errors that may affect the accuracy of mortality estimates [3, 9]. For example, mothers may not report some births/deaths (particularly if they have occurred several years prior to the survey), may misreport the child’s age or age at death [10, 11], or may misclassify stillbirths as neonatal deaths and vice-versa [12]. Despite these potential issues, FPH and FBH constitute the current best practice in mortality measurement in LMICs with limited CRVS systems.

Unfortunately, retrospective interviews like FPH and FBH also do not permit the measurement of mortality in real time, because household surveys are often only conducted every 3–5 years in most LMICs [13]. Furthermore, these surveys often have limited sample size, and only allow estimation of average mortality rates for a period of several years before the survey.

The Real-Time Monitoring of Under-Five Mortality (RMM) project aimed to address this lack of timely data by testing alternative approaches to measuring mortality on a monthly basis. By collecting data continuously, several of the RMM methods may also alleviate concerns about sample selection biases and reporting errors that affect other data sources. RMM methods included: community-based reporting on vital events, various adjustments to HMIS data, as well as rapid household survey methods (e.g., summary birth histories). They were tested in several Sub-Saharan African countries [14], with heterogeneous results [15]. In Ethiopia and Malawi, for example, community-based reports of vital events collected by paid workers under-estimated mortality levels compared to survey data [16–18]. In Mali, on the other hand, unpaid community-based workers collected mortality data that yielded estimates consistent with survey data [19]. In Malawi, adjustments to health facility data on mortality resulted in severe under-estimates of under-5 mortality rates. Finally, rapid household survey methods also failed to produce reliable estimates of under-5 mortality.

In this paper, we report results from the implementation of the RMM project in Ghana. In that country, estimates of child under-5 mortality are produced using a number of different data sources. Several household surveys have been conducted, including most recently the 2014 DHS. This survey yielded a nationwide estimate of the under-5 mortality rate of 60 deaths per 1,000 live births for the period 2010–2014. It also indicated large regional variations in under-5 mortality, with mortality risks more than twice as high in the Northern region of Ghana than in the greater Accra region where the capital city is located. But such household surveys are only conducted every 3–5 years in Ghana, and thus do not permit monitoring mortality continuously.

Several health and demographic surveillance systems (HDSS) also provide alternative estimates of under-5 mortality in Ghana. HDSS are large open cohorts, which monitor demographic trends continuously in small, well-delineated populations (e.g., 10,000–150,000 individuals). They do so through regular household visits (e.g., 3–4 times per year) during which an interviewer records changes to household membership that may have occurred through birth, death or migration. In Ghana, the three HDSS are located throughout the country, in the greater Accra [20], Brong-Ahafo [21] and Upper East [22] regions. HDSS data may not be representative of recent mortality trends in Ghana, however, because 1) each HDSS location was purposefully selected, and 2) HDSS are often the setting of clinical and health systems trials, which may affect mortality levels.

The Ghana Births and Deaths Registry (GBDR) oversees Ghana’s CRVS system. CRVS data may help produce estimates of under-5 mortality rates on an annual basis, by dividing the number of deaths to children under 5 and the number of births recorded in a year. But in practice, the registration coverage for both births and deaths is too low to enable such measurement in Ghana. In 2014, only 2/3 of births were registered and 25% of all deaths were registered in the country [23]. In addition, these events may be registered with significant delays, thus potentially postponing the availability of accurate vital statistics.

The RMM project was designed to help increase the registration of events by the BDR, and to produce interim estimates of under-5 mortality rates. It entailed the training and deployment of community-based volunteers (CBVs) to record vital events, and promote registration of events with the BDR, in several districts of the Northern Region of Ghana. If successful, this approach to collecting mortality data continuously could be more rapidly scaled-up to other districts of the country than other approaches like HDSS. In this paper, we assessed the completeness and timeliness of the records collected by CBVs. This provides an initial assessment of the capacity of this RMM method to produce real-time mortality estimates. We then evaluated the ability of CBV data to produce accurate mortality estimates, through comparison with FPH data collected in the same communities.

## Methods

### Setting

The RMM project was implemented in the Northern Region of Ghana, which has the highest total fertility rate and under-five mortality rate of any region in the country [24]. The project was a partnership between the Institute for International Programs at Johns Hopkins University (IIP-JHU), the Noguchi Memorial Institute for Medical Research (NMIMR) at the University of Ghana, and the GBDR. The GBDR was the primary government partner, with IIP-JHU and NMIMR providing technical support for project implementation.

Three districts of the Northern Region were selected for RMM implementation: Tolon/Kumbugu (Tolon hereafter), Karaga, and Zabzugu/Tatale (Zabzugu hereafter). According to the 2010 census, the combined population of these districts was 313,891. The district selection process was purposive and focused on some of the most rural and deprived areas of the region. The location of the study districts is shown in S1 File, which also provides details on the sampling process. In short, within each study district, we sampled at random with probability proportionate to size approximately 20 census enumeration areas (EA). Then from this selection of EAs, we formed RMM study clusters. In EAs that included multiple small population settlements, each separate village formed a distinct RMM cluster. In other EAs that coincided with one locality, the entire EA became a single RMM cluster. Finally, in larger and possibly more urban communities, several EAs were joined together to form a cluster. In those larger clusters, several CBVs usually collected birth and death reports, whereas only one CBV worked in each of the smaller clusters described above. In total, there were 36 RMM clusters in Karaga, 28 in Tolon, and 32 in Zabzugu. They were formed from 20, 21 and 20 EAs in each of these districts, respectively). The complete sampling procedures are also described in S1 File. Together the 96 RMM clusters covered an estimated population of 36,661 in 2010.

### Implementation of the RMM method

One objective of the RMM project was to improve the registration of births and deaths by the GBDR. To do so, it relied on CBVs engaged in each study cluster to register pregnancies, births and deaths among children under five. The RMM project was nested within the organizational structure of the GBDR in order to facilitate the potential scale-up of the RMM data collection strategy to other districts in Ghana. GBDR officers at national, regional, and district levels, were thus involved in coordinating and supervising CBV activities. Research assistants from NMIMR also assisted GBDR officers in monitoring and supervision of CBVs.

After selecting the study clusters, we carried out community engagement activities and liaised with community leaders. During that stage, we selected CBVs in the sampled clusters. CBVs were community members recruited to collect and report vital event data in their respective cluster. They were not paid by the project. They were recruited based on criteria including literacy, community acceptance, previous work as a volunteer or in community-based programs, and willingness to work in the cluster over the period of the RMM project. In clusters where candidate CBVs of both sexes were available, we selected women rather than men. In some clusters where no literate CBVs were available on a full-time basis, we asked community leaders to select a schoolteacher or another active community member to serve as an informant.

We then participated in a series of community meetings (known as *durbars*) during which we explained the project, emphasized the importance of registration of vital events, and introduced CBVs and their new role to community members. During the durbars, GBDR officers issued birth certificates to unregistered children less than one year of age to raise birth registration rates and foster community interest and engagement.

The selected CBVs from each district were trained over a three-day period in Tamale, Northern Region. They learned to 1) conduct surveillance of pregnancies, births, and deaths; 2) complete vital events registers; 3) complete monthly summary forms (MSF) for reporting vital events to GBDR supervisors; and 4) complete official birth and death registration forms. Evaluations conducted at the end of training showed large heterogeneity in the competencies achieved by CBVs. GBDR officers were encouraged to supervise more frequently the CBVs with the lowest command of the reporting tasks at the end of training.

CBV data collection consisted of identifying and recording vital events (pregnancies, births, and under-5 deaths) through passive and active detection. Active detection entailed monthly visits to each household in the study cluster to ask about the recent occurrence of pregnancies, births, and deaths. CBVs were also encouraged to follow up on all pregnancies they had previously registered, and to consult traditional birth attendants, imams, chiefs, priests, or any other person who might be aware of new births or deaths in the community. Passive detection consisted of community members spontaneously contacting CBVs to notify them of vital events.

The CBVs recorded each detected event in specific registers (e.g., pregnancy register). Then they completed an MSF, in which they transferred all recorded pregnancies, births, and deaths in their catchment area for each respective month. Finally, MSF were transmitted to district GBDR supervisors and study supervisory staff for collation and forwarding to the study office in Accra. An example MSF is available in S2 File.

The GBDR (with assistance from IIP-JHU and NMIMR) held quarterly data review meetings with the CBVs to review monthly reports, provide refresher training, resolve implementation problems, and collect outstanding reports. The meetings also served as an incentive to the CBVs by providing opportunities to gather with peers and receive travel allowances. Research assistants from NMIMR entered data from the event registers and MSF in an Excel spreadsheet. These CBV databases were then cleaned at the NMIMR office, and analyzed using Stata versions 12 and 13. After an initial three-month pilot period, CBV data collection lasted for 21 months, from January 2012 to October 2013.

### Validation of the RMM method

We tested the hypothesis that data on vital events recorded by CBVs would yield estimates of mortality rates among children under five that are as accurate as the current best practice of mortality measurement, i.e., collecting mortality data during retrospective interviews with women of reproductive ages. To do so, we used data from FPH as a reference, against which mortality rates calculated from CBV data were compared. FPH data were collected during a census of the study clusters conducted at the end of the project. They were collected in the exact areas where CBVs collected mortality data themselves. But the data collectors who collected FPH data were not the CBVs themselves. This ensured that they were not aware beforehand that a birth/under-5 death may have occurred in the households they visited. The questionnaires used during this census are included in S3 File.

During the census, every woman aged 15–49 years residing in one of the study clusters was asked to complete an interview, which included a FPH. FPHs started by enumerating all the pregnancies a woman had ever had, in chronological order, starting from the first pregnancy. For each reported pregnancy, respondents were asked to state whether the pregnancy ended in a live birth or in another outcome (e.g., abortion, miscarriage, or stillbirth). Then, for each live birth, respondents were asked to report a) the date of birth of the child; b) the sex of the child; c) whether the child was a singleton or part of a multiple birth (e.g., twins); and d) whether the child was still alive. Finally, if a live-born child had died, respondents were asked how old the child was when s/he died.

FPHs were collected using computer-assisted personal interviewing (CAPI) technology by trained interviewers. The interview also included questions about a) maternal health for each live birth of the past two years prior to data collection, and b) birth (and possibly death) registration for each live birth of the past 5 years prior to data collection. In addition to controls and checks programmed in the CAPI system, careful training and field supervision of interviewers were used to try to minimize errors known to occur during the collection of FPH data, e.g., errors in age and date declaration, misclassifications of stillbirths as neonatal deaths (and vice-versa), and omission of births and deaths [9, 10, 25, 26]. As mentioned elsewhere [14], there were initial concerns about the quality of the FPH data collected in Ghana. In this paper, we thus report extensive analyses of potential errors and inconsistencies in such data (see below).

### Ethical approval

The Institutional Review Boards at the Johns Hopkins Bloomberg School of Public Health and NMIMR approved the RMM project protocol in July 2011 and September 2011, respectively. Oral consent was obtained from all participants.

### Data analysis

We first described the characteristics of recruited CBVs, and we assessed the quality of data collected by these CBVs. We measured delays in reporting of events by CBVs by comparing the date of an event to the date that event was actually reported. We also tested whether sex ratios at birth in CBV data a) departed from expected values (e.g., 102–107 male births for each 100 female births [27]); and b) varied by district. Finally, we examined the ratios of reported neonatal and infant deaths to under-five deaths.

Second, we evaluated the quality of our reference dataset, i.e., the FPH data collected during the end-of-project census. Similar to the CBV data, we calculated the sex ratios at birth and the ratios of reported infant and neonatal deaths to under-five deaths. This was done to assess whether FPH data departed from the ranges of sex ratio and rate ratios observed in other populations [3]. We also investigated possible displacements of births and deaths in the FPH data, and misclassifications of stillbirths as neonatal deaths (and vice-versa).

Finally, we compared CBV and FPH estimates of mortality rates. To do so, we computed neonatal, infant, and under-five mortality rates in each dataset, by dividing the number of such deaths reported in a specific period by the total number of births for the same period. These measures correspond to conventional calculations of mortality rates, even though they are not strictly cohort or period life-table measures. They have been used in other validation studies of RMM strategies for data collection [15–17, 28].

We assessed the equivalence of mortality estimates from the CBV and FPH data by computing the ratio of both rates, then multiplying by 100. Both the CBV and FPH datasets were collected from censuses of the study clusters, but the selection of study clusters entailed random sampling within districts. We thus computed approximated standard errors and confidence intervals for all rates and ratios by the bootstrap method [29].

For each of the analyses described above, we computed estimates separately by district (or by grouping of districts), before computing a combined estimate for all districts (“combined” analysis thereafter). We conducted a district-level analysis for two reasons. First, the supervision of CBV was implemented at the district level by the GBDR. As a result, the accuracy of CBV estimates, as well as the timeliness of CBV data may vary from district to district. Second, in Ghana, decision-making over health systems resources has been decentralized to the district level [30, 31]. As a result, district management teams (DHMTs) require accurate district-level data to make decisions and evaluate their activities.

We also conducted each analysis for different time periods: from January to December 2012, from October 2012 to September 2013, and for the entire duration of the RMM project (January 2012 to September 2013). Finally, we compared the estimated mortality rates to estimates obtained from the 2014 Ghana DHS for the Northern region. We computed DHS estimates of those rates for the same time period and using the same methodology used for the analysis of CBV and FPH data.

## Results

### Implementation of the CBV approach

In total, we recruited 125 CBVs, of whom 116 remained employed in January 2013 (Table 1). We faced retention issues in Karaga, where 9 out of 41 recruited CBVs (21.9%) had dropped out of the RMM project by January 2013, whereas in other districts all CBVs remained in their post. Despite our emphasis on recruiting women, 97.9% of the CBVs we recruited were men. CBVs were primarily young adults: 73.3% of all CBVs were aged less than 35 years old. But CBVs were younger in Zabzugu than in the other districts: in that district, 89.3% of CBVs were less than 35 years old, as compared to 54.0% in Tolon and 71.9% in Karaga. In a number of communities in all three districts, we were unable to identify a functionally literate individual to serve as the CBV. Approximately 5% of all CBVs had thus never been to school. The difficulties in recruiting literate CBVs were greater in Karaga, where close to one in six CBVs never went to school. Finally, there were differences in levels of prior experience among recruited CBVs between the three districts. Whereas in Zabzugu and Tolon, more than half of all CBVs had prior experience in recording vital events, only 28.1% of CBVs had such experience in Karaga.

**Table 1:** Distribution of CBV characteristics by district.

	District			
	Zabzugu	Tolon	Karaga	Total
Total CBVs originally recruited (n)	47	37	41	125
Total CBVs engaged in RMM as of January 2013* (n)	**47**	**37**	**32**	**116**
**Male (%)**	97.9	100.0	96.9	98.3
**Age (%)**				
<25	40.4	13.5	21.9	26.7
25–34	48.9	40.5	50.0	46.6
35–44	8.5	35.1	9.4	17.2
45+	2.1	10.8	18.8	9.5
**Level of Education (%)**				
None	0.0	5.4	12.5	5.2
Primary	6.4	0.0	6.3	4.3
Middle School	27.7	35.1	28.1	30.2
High School +	63.8	45.9	40.6	51.7
Other	2.1	13.5	12.5	8.6
**Previous Volunteer Experience (%)**	59.6	70.3	50.0	60.3
**Previous experience in recording birth and death data (%)**	57.4	51.4	28.1	47.4

Notes

* As of the beginning of 2013, RMM was supporting the work of 116 total CBVs. Reasons for changes from the CBVs originally recruited include: CBVs had significant problems filling out the forms and needed to be replaced (2); CBV was illiterate and was not supported by a literate CBV, so RMM support was discontinued (4); CBV left for school (3); CBV left because they considered the workload too heavy (3); CBV left due to personal issues (1); CBV passed away (1). Five of the 14 CBVs who left were replaced; nine were not.

During the course of the project, CBVs in Tolon and Zabzugu compiled and submitted MSF consistently each month (100% of communities reporting), but CBVs in Karaga reported data less consistently. This was particularly so for the more remote areas of the district, and during the rainy season. As a result, only 80% of the Karaga communities reported data on births and deaths during August and September 2013.

### Quality of the CBV data

**Fig 1:**
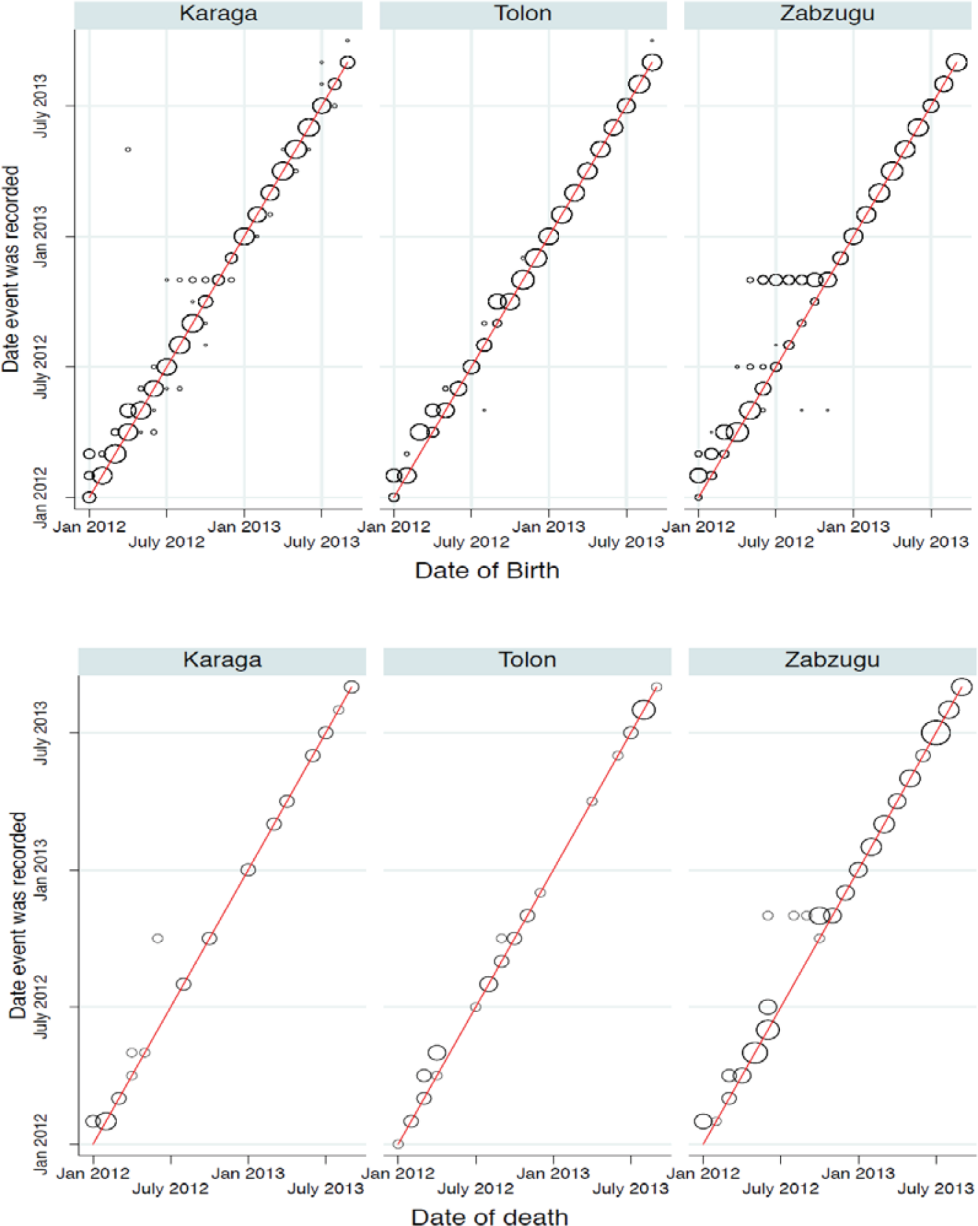
Reporting delays in CBV data. **Panel a: delays in reporting of births. Panel b: delays in reporting of deaths, by district.** *Notes*: *each circle represents the number of events having occurred during month* x *and being recorded during month y*. *The size of each circle is proportional to the number of such events*. *The red line represents the absence of reporting delays*, *i*.*e*., *x = y*. *Circles above the red line represent events reported with delays*, *whereas circles below the red line represent events that were reported before they occurred (i*.*e*., *possible data entry errors)*.

In all three districts, both births and deaths were reported with delays in the first months of 2012 (Fig 1A and 1B). In Karaga and Zabzugu, CBVs appear to have cleared a significant backlog of unreported events in November 2012 (Fig 1A). In that month, they reported events dating back to May 2012 (Zabzugu) and July 2012 (Karaga). After November 2012, the reporting delays declined, particularly in Zabzugu. Some events also appear to have been reported prior to their occurrence (i.e., events below the diagonal line in Fig 1A and 1B). However, such events were rare (e.g., n = 29 births) and may have been due to data entry errors. Removing them from the analysis did not affect the results from the assessment of CBV data. The reporting of deaths followed the same pattern (Fig 1B). In Zabuzugu, no deaths were reported between August 2012 and October 2012, but the deaths that occurred during those months were then reported in November 2012. The delays in reporting events were the shortest in Tolon.

CBVs reported 2,819 births, with 719, 1,001, and 1,099 births reported in Karaga, Tolon, and Zabzugu, respectively (Table 2). They also reported 137 under-five deaths with Karaga, Tolon, and Zabzugu reporting 29, 33, and 75 deaths respectively. There were 55 deaths among neonates reported by CBVs during the course of the study, and 29 deaths reported among infants aged 1–11 months (post-neonatal deaths).

**Table 2:** Assessments of the quality of CBV data, by district and study period.

	Births					Deaths				
	Number of births reported				Sex Ratio at Birth^a^	Number of deaths reported			Age patterns of under-5 mortality	
Period	Total	Males	Females	Missing		Neonatal^b^	Infant^c^	Under-5^d^	Neonatal/ Under-5	Infant/ Under-5
Karaga										
01/2012-12/2012	431	229	200	2	114.5	7	11	16	43.8	68.8
10/2012-09/2013	341	178	159	4	111.9	5	9	15	33.3	60.0
Entire RMM period	719	376	337	6	111.6	11	19	29	37.9	65.5
Tolon										
01/2012-12/2012	559	287	272	0	105.5	8	13	23	34.8	56.5
10/2012-09/2013	617	343	271	3	126.6	8	12	15	53.3	80.0
Entire RMM period	1,001	536	462	3	116.0	14	21	33	42.4	63.6
Zabzugu										
01/2012-12/2012	600	317	276	7	114.9	19	28	39	48.7	71.8
10/2012-09/2013	627	353	270	4	130.7	17	27	47	36.2	57.4
Entire RMM period	1,099	600	492	7	121.9	30	44	75	40.0	58.7
Combined										
01/2012-12/2012	1,590	833	748	9	111.4	34	52	78	43.6	66.7
10/2012-09/2013	1,585	874	700	4	124.9	30	48	77	39.0	62.3
Entire RMM period	2,819	1,512	1,291	16	117.1	55	84	137	40.1	61.3

Notes

^a^ sex ratios at birth are defined as the number of male births for every 100 female births. A sex ratio at birth over 100 indicates that there are more male than female births; whereas a sex ratio at birth under 100 indicates the opposite.

^b^ The count of neonatal deaths includes all deaths among live-born children having occurred less than 28 days after birth.

^c^ the count of infant deaths includes all deaths among live-born children having occurred less than one year after birth. The count of infant deaths includes the count of neonatal deaths.

^d^ The count of under-5 deaths includes all deaths having occurred less than 5 year after births. It includes the number of neonatal and infant deaths.

The CBV data indicated elevated sex ratios at birth across all study districts. Overall, there were 117.1 male births recorded for each 100 female births recorded. The corresponding figures were 111.6, 116.0, and 121.9 in Karaga, Tolon, and Zabzugu, respectively. Sex ratios at birth were highest in Tolon and Zabzugu during the period from October 2012 to the end of the project: for example, during that period, the sex ratio at birth in Zabzugu was 130.7, as compared to 114.9 during the period from January 2012 to December 2012.

Table 2 also presents the age patterns of under-5 mortality according to CBV data. The ratios of neonatal to under-five deaths ranged from 33.3% in Karaga (between October 2012 and September 2013) to 53.3% in Tolon (during the same period), whereas the ratios of infant to under-five deaths ranged from 56.5% in Tolon (between January and December 2012), to 80.0% in Tolon (between October 2012 and September 2013).

### Quality of the FPH data

During the end-of-project census, 2,835 births were reported through FPH to have occurred between January 2012 and September 2013. Among those, respondents in Karaga, Tolon, and Zabzugu reported 809, 943, and 1,083 births, respectively (Table 3). FPH respondents reported that 208 under-five deaths occurred during that period across all three districts; 96 of these deaths were reported to have occurred in Zabzugu, whereas 69 occurred in Tolon, and 43 occurred in Karaga.

**Table 3:** Assessments of the quality of FPH data, by district and study period.

	Births					Deaths				
	Number of births reported				Sex Ratio at Birth^a^	Number of deaths reported			Age patterns of under-5 mortality	
Period	Total	Males	Females	Missing		Neonatal^b^	Infant^c^	Under-5^d^	Neonatal/ Under-5	Infant/ Under-5
Karaga										
01/2012-12/2012	441	215	226	—	95.1	6	12	26	23.1	46.2
10/2012-09/2013	465	232	233	—	99.6	10	15	26	38.5	57.7
Entire RMM period	809	398	411	—	96.8	15	23	43	34.9	53.5
Tolon										
01/2012-12/2012	501	270	231	—	116.9	13	21	41	31.7	51.2
10/2012-09/2013	598	327	271	—	120.7	10	19	39	25.6	48.7
Entire RMM period	943	509	434	—	117.3	22	35	69	31.9	50.7
Zabzugu										
01/2012-12/2012	605	349	256	—	136.3	10	19	54	18.5	35.2
10/2012-09/2013	657	363	294	—	123.5	10	24	54	18.5	44.4
Entire RMM period	1,083	608	475	—	128.0	17	38	96	17.7	39.6
Combined										
01/2012-12/2012	1,547	834	713	—	117.0	29	52	121	24.0	43.0
10/2012-09/2013	1,720	922	798	4	115.5	30	58	119	25.2	48.7
Entire RMM period	2,835	1,515	1,320	—	114.8	54	96	208	26.0	46.2

Notes

^a^ sex ratios at birth are defined as the number of male births for every 100 female births. A sex ratio at birth over 100 indicates that there are more male than female births; whereas a sex ratio at birth under 100 indicates the opposite.

^b^ The count of neonatal deaths includes all deaths among live-born children having occurred less than 28 days after birth.

^c^ the count of infant deaths includes all deaths among live-born children having occurred less than one year after birth. The count of infant deaths includes the count of neonatal deaths.

^d^ The count of under-5 deaths includes all deaths having occurred less than 5 year after births. It includes the number of neonatal and infant deaths.

The FPH data showed reported sex ratios at birth that ranged from 116.9 to 136.3 male births for 100 female births in Zabzugu and Tolon. In Karaga, they ranged from 95.1 to 99.6. The ratios of neonatal deaths to under-five deaths also varied across districts. Over the entire duration of the RMM project, they ranged from 17.7% in Zabzugu to 34.9% in Karaga. The ratios of infant deaths to under-five deaths showed similar patterns, with the highest ratios observed in Karaga and Tolon. Additional assessments of the quality of FPH data are provided in S4 File.

### Comparison of CBV and survey data

The comparison of Tables 2 and 3 show important discrepancies between the two data sources. First, whereas in Tolon and Zabzugu, both data sources indicated elevated sex ratios at birth, this was not the case in Karaga. In that district, the CBV data displayed a high sex ratio at birth, whereas the FPH data yielded estimates of the sex ratios at birth below 100. Second, the CBV data yielded estimates of the age patterns of mortality that differed from those estimated from FPH data. In all districts, CBV records suggested that a larger proportion of all under-five deaths occurred among neonates and infants than the FPH data. This was particularly so in Zabzugu, where 40.0% of all under-five deaths occurred among neonates according to CBV data, as compared to 17.7% according to FPH data (Tables 2 and 3).

Tables 4, 5 and 6 further describe the level of agreement in estimated rates of neonatal, infant, and under-five mortality between the CBV and FPH datasets, respectively. For these analyses, we combined data from Karaga and Tolon due to limited sample sizes and similar patterns of CBV reporting in each district.

**Table 4:** Under-five mortality rates estimated from CBV records and FPH data and corresponding 95% confidence intervals.

	CBV Data		FPH Data		Ratio CBV/FPH	
Period	Rate	95% CI	Rate	95% CI	%	95% CI
Karaga/Tolon						
01/2012-12/2012	39.4	(27.0, 51.8)	74.3	(56.5, 92.1)	53.0	(31.7, 74.4)
10/2012-09/2013	31.3	(19.6, 43.0)	58.3	(43.2, 73.5)	53.7	(28.8, 78.5)
Entire RMM period	36.0	(26.9, 45.2)	65.1	(52.9, 77.3)	55.4	(37.3, 73.5)
Zabzugu						
01/2012-12/2012	65.0	(44.2, 85.8)	82.6	(58.6, 106.4)	78.7	(43.1, 114.1)
10/2012-09/2013	75.0	(53.1, 96.9)	82.2	(59.3, 105.1)	91.2	(52.8, 129.6)
Entire RMM period	68.2	(52.1, 84.4)	85.9	(67.6, 104.1)	79.5	(54.2, 104.7)
Combined						
01/2012-12/2012	49.1	(37.8, 60.3)	77.6	(63.4, 91.8)	63.2	(44.5, 82.0)
10/2012-09/2013	48.6	(37.9, 59.3)	67.4	(54.8, 80.1)	72.0	(51.0, 93.1)
Entire RMM period	48.6	(40.3, 56.8)	73.0	(63.0, 83.0)	66.6	(51.7, 81.4)

Notes: Rates are per 1,000 live births. Confidence intervals were obtained by bootstrapping standard errors (2,000 bootstrap samples). Ratios CBV/survey less than 100 indicate that CBV data under-estimate the rate of interest relative to FPH data. Ratios above 100 indicate the opposite. For the ratio CBV/FPH, confidence intervals that include 100 indicate that we cannot reject the hypothesis of statistical equivalence between the two estimated rates.

**Table 5:** Infant mortality rates estimated from CBV records and FPH data and corresponding 95% confidence intervals.

	CBV Data		FPH Data		Ratio CBV/FPH	
Period	Rate	95% CI	Rate	95% CI	%	95% CI
Karaga/Tolon						
01/2012-12/2012	24.2	(14.4, 34.1)	35.0	(22.9, 47.2)	69.2	(29.7, 108.7)
10/2012-09/2013	21.9	(12.2, 31.6)	32.0	(21.0, 42.9)	68.5	(28.5, 108.6)
Entire RMM period	23.3	(15.9, 30.7)	33.1	(24.2, 42.0)	70.2	(40.3, 100.2)
Zabzugu						
01/2012-12/2012	46.7	(29.1, 64.2)	31.4	(17.2, 45.7)	148.6	(49.4, 247.8)
10/2012-09/2013	43.1	(27.0, 59.2)	36.5	(21.4, 51.6)	117.9	(42.4, 193.4)
Entire RMM period	40.0	(28.1, 51.9)	35.1	(23.8, 46.3)	114.1	(59.1, 169.1)
Combined						
01/2012-12/2012	32.7	(23.8, 41.6)	33.6	(24.3, 42.9)	97.3	(57.4, 137.2)
10/2012-09/2013	30.3	(21.5, 39.0)	33.7	(24.9, 42.5)	89.8	(53.8, 125.8)
Entire RMM period	29.8	(23.2, 36.4)	33.9	(26.8, 40.9)	88.0	(59.9, 116.0)

Notes: Rates are per 1,000 live births. Confidence intervals were obtained by bootstrapping standard errors (2,000 bootstrap samples). Ratios CBV/survey less than 100 indicate that CBV data under-estimate the rate of interest relative to FPH data. Ratios above 100 indicate the opposite. For the ratio CBV/FPH, confidence intervals that include 100 indicate that we cannot reject the hypothesis of statistical equivalence between the two estimated rates.

**Table 6:** Neonatal mortality rates estimated from CBV records and FPH data and corresponding 95% confidence intervals.

	CBV Data		FPH Data		Ratio CBV/FPH	
Period	Rate	95% CI	Rate	95% CI	%	95% CI
Karaga/Tolon						
01/2012-12/2012	15.2	(7.4, 22.9)	20.2	(10.9, 29.4)	75.1	(16.2, 134.1)
10/2012-09/2013	13.6	(6.2, 20.9)	18.8	(10.5, 27.1)	72.1	(14.0, 130.2)
Entire RMM period	14.5	(8.8, 20.3)	21.1	(14.1, 28.1)	68.8	(31.5, 106.1)
Zabzugu						
01/2012-12/2012	31.7	(17.2, 46.1)	16.5	(6.1, 26.9)	191.6	(-30.8, 413.9)
10/2012-09/2013	27.1	(14.4, 39.8)	15.2	(5.8, 24.7)	178.1	(-36.3, 392.5)
Entire RMM period	27.3	(17.3, 37.3)	15.7	(8.3, 23.1)	173.9	(44.1, 303.7)
Combined						
01/2012-12/2012	21.4	(14.0, 28.8)	18.7	(11.8, 25.7)	114.1	(52.4, 175.7)
10/2012-09/2013	18.9	(12.0, 25.8)	17.4	(11.2, 23.7)	108.5	(48.1, 168.9)
Entire RMM period	19.5	(14.2, 24.9)	19.0	(13.9, 24.2)	102.4	(63.5, 141.4)

Notes: Rates are per 1,000 live births. Confidence intervals were obtained by bootstrapping standard errors (2,000 bootstrap samples). Ratios CBV/survey less than 100 indicate that CBV data under-estimate the rate of interest relative to FPH data. Ratios above 100 indicate the opposite. For the ratio CBV/FPH, confidence intervals that include 100 indicate that we cannot reject the hypothesis of statistical equivalence between the two estimated rates.

The CBV estimate of the under-five mortality rate was consistently lower than the rate estimated from FPH data (Table 4). In Karaga/Tolon, the CBV data yielded an estimate of the under-five mortality rate of 36 deaths per 1,000 over the entire course of the RMM project, as compared to 65.1 according to the FPH data. The confidence interval for the ratio of these two rates (ratio = 55.4) did not include the possibility of statistical equivalence between the two rate estimates (37.3 to 73.5). For Zabzugu, the CBV data yielded an estimate of 68.2 deaths per 1,000 vs. 85.9 per 1,000 according to the FPH data, but the confidence interval of the ratio of these two rates did not rule out the possibility of statistical equivalence (ratio = 79.5, 95% Confidence interval = 54.2 to 104.7). When data from all districts were combined, we found that the under-five mortality rate estimated from CBV data was 33% lower than the estimate from FPH data. For comparison, the estimated rate of under-5 mortality in the northern region in 2012–2013 according to the 2014 Ghana DHS was 76.3 per 1,000 (95% CI = 50, 9 to 109.0), i.e., comparable to the FPH estimate (73.0 deaths per 1,000).

The patterns of concordance between the two datasets were more complex when focusing on neonatal and infant mortality rates. In the combined analyses, there was significant agreement between the two datasets for these two rates. The CBV estimate of the infant mortality rate (Table 5) was 29.8 deaths per 1,000 live births vs. 33.9 per 1,000 according to the FPH data (ratio = 88.0, 95% CI = 59.9, 116.0). The CBV estimate of the neonatal mortality rate (Table 6) was 19.5 deaths per 1,000 live births vs. 19.0 according to the FPH data (ratio = 102.4, 95% CI = 63.5, 141.4). However, when disaggregated by district, the CBV estimates of infant and neonatal mortality rates in Karaga/Tolon were lower than those obtained from FPH data, whereas the CBV estimates were higher than FPH estimates in Zabzugu. For example, the ratio of the CBV and FPH estimates of the infant mortality rate was 68.8 (95% CI = 31.5, 106.1) in Karaga/Tolon, but it reached 173.9 (95% CI = 44.1, 303.7) in Zabzugu.

## Discussion

In this paper, we report on our test of a real-time mortality monitoring system in highly impoverished rural areas of Ghana. This system relied on CBVs to continuously record and report each pregnancy, birth, and under-five death occurring in the community to which they were assigned. Our assessment of the quality of the data collected by CBVs over a 21-month period indicated that this approach generated biased mortality estimates. In particular, under-five mortality rates estimated from CBV data were significantly lower than estimates obtained from reference FPH data collected in the same study clusters.

Our assessment of the quality of CBV data has several limitations. First, it is based on a limited number of events in each district. As a result, we had to pool data from two districts in conducting selected analyses. Due to limited sample sizes, our assessment of the mortality estimates from CBV data was also conducted over an aggregated 21-month period, rather than on a month-by-month basis. It thus does not constitute a proper test of the capacity of the RMM method to produce real-time mortality estimates. Finally, we could not test for improvements in reporting parameters over time, nor could we investigate the correlates of reporting delays (e.g., ethnicity or age of the mother, parity of the child).

Second, the FPH data did not have the exact same sampling universe as the CBV data. Some women who were residents of the study communities at the time of FPH data collection may have been residents of other communities at the time when they gave birth. Similarly, some women who gave birth in the study communities during the course of CBV data collection may have migrated out of the study areas before the beginning of FPH data collection. In some instances, children may also be fostered (out-migration) to another relative’s home outside of the study community (e.g., a grandparent, or an uncle), whereas other children who were born outside of the study communities may rapidly come to live with a local relative (i.e., in-migration). Some births may thus have been included in only one of the two data sources, creating possible discrepancies between estimates. More precise assessments of the CBV data would require record linkages with FPH at the individual level to control for differences in sampling universe between datasets [32].

Third, even though it yielded estimates of under-five mortality comparable to those from the 2014 Ghana DHS, our reference dataset (FPH) was affected by sample selection biases and reporting errors. These included imbalanced sex ratios at birth, and age and date heaping (see S4 File). For example, in 2 out of 3 districts, the FPH data yielded estimates of the sex ratio at birth that were well above the normal range of 102–107 male births for every 100 female births [27]. In the third district, the estimated sex ratio at birth was below this range. Whereas the 2008 DHS and 2011 MICS datasets also indicated elevated sex ratios at birth in the northern region of Ghana, such deviations were not seen in the 2014 Ghana DHS. Some births may thus not have been reported during the FPH collected as part of this study. If the births of children who subsequently died were more likely to be omitted during FPH [10], then FPH data may have under-estimated under-five mortality in the study clusters. However, since under-5 mortality estimates from CBV data were below those obtained from FPH, our conclusions that CBV estimates are biased downwards remain unaffected by this issue.

The downward bias in mortality estimates obtained from CBV data was less pronounced for neonatal/infant mortality rates than for under-five mortality rates (Tables 5 and 6). In one district (Zabzugu), we even found neonatal and infant mortality rates that were *higher* than those estimated from FPH data. This pattern suggests that CBVs were more likely to miss the deaths of children aged 1–4 years old than deaths of neonates and infants. It also indicates that in some settings (e.g., Zabzugu), CBVs may elicit more complete data on neonatal and infant deaths than FPH interviews, which may be affected by omissions of births that resulted in a neonatal death, misreporting of ages at death, and/or misclassifications of neonatal deaths as stillbirths [3, 10].

A closer examination of the RMM datasets collected in other countries suggests that pregnancy monitoring may be a key reason why CBVs collect relatively higher numbers of neonatal and infant deaths. Indeed, in the other RMM countries (Ethiopia, Mali, and Malawi) where community-based workers inquired about pregnancies during household visits and surveillance activities, we often found higher estimates of neonatal and infant mortality in the CBV data than in the reference FPH data [17, 18, 33]. Pregnancy monitoring may thus be a key component of a vital registration system that accurately measures neonatal and infant mortality.

CBVs may also be more effective in recording neonatal and infant deaths than FPH interviewers, because they are community members, who may hear about such events through social and conversational networks [34, 35]. FPH interviewers, on the other hand, are most commonly outsiders and strangers to whom respondents may be reluctant to disclose such private, emotional events [36, 37].

Our assessment of the CBV data collection system has several other important implications. On the one hand, it highlights the importance of conducting tests of new data collection systems across multiple administrative units in LMICs. This is especially important because the qualifications of data collectors, or the level of their supervision, may vary significantly across administrative units. Single-site validation studies, or studies that systematically pool data from multiple administrative units, risk misrepresenting the potential benefits of new data collection systems. For example, in our study, the combined dataset suggested that CBVs recorded approximately the same number of births as the FPH data (2,819 vs. 2,835). But this congruence masked significant discrepancies at the district level.

On the other hand, our results also suggest that CBV systems may not yield reliable estimates of mortality trends. Further operational research is needed to a) generate a comprehensive understanding of the systemic factors that influence the operations of CBVs in vital events monitoring, and b) improve the completeness and accuracy of the data CBVs generate. This may include, for example, testing the use of mobile phones and other communication technologies to accelerate the reporting of events [38]. It may also entail testing additional strategies of community mobilization, or the provision of incentives to community residents for the registration of vital events. Finally, it may require developing techniques to ensure the timely reporting of all pregnancies. Even though >90% of women attend antenatal care services in Ghana [24], some women may still be reluctant to disclose a pregnancy during CBV visits, at least during the first trimester.

Because the coverage of vital registration systems in LMICs, particularly death registration, remains very low, monitoring trends in child mortality rates over the next 15 years will require multiple efforts. Where possible, new data collection systems should be tested and rolled out, which permit monitoring trends in mortality in (near) real-time. These systems could include, for example, sample vital registration systems [39, 40] or health and demographic surveillance systems [41]. They should also help develop the basis for a universal vital registration system in targeted LMICs [39, 41, 42]. In addition, improvements in FPH and other survey data on mortality are needed to ensure that estimates of neonatal, infant, and child mortality are accurate in settings where no other data collection systems are available.

## Supporting information

S1 File Selection of RMM project areas and sampling. (PDF)

S2 File Sample monthly summary form (MSF). (PDF)

S3 File Study questionnaires (end-of-project census). (PDF)

S4 File Assessment of the quality of FPH data. (PDF)
