# Supplementary material for: Using community-based reporting of vital events to monitor child mortality: Lessons from rural Ghana
Source: PLoS One. 2018 Jan 30;13(1):e0192034. doi: 10.1371/journal.pone.0192034 (PMC5790256; doi:10.1371/journal.pone.0192034)
Supplement: S1 File — (PDF) [file pone.0192034.s001.pdf]

# Supporting Information File 1: Selection of RMM project areas and sampling

In collaboration with GHS and BDR leadership, we purposefully selected RMM areas in the most rural parts of some of the poorest areas in the Northern Region (Figure A1.1). The Northern Region has both the highest total fertility rate and the highest under-five mortality rate among regions in Ghana. Some general characteristics for the country as a whole, as well as the Northern Region, the aforementioned sampled districts, and the specific RMM sample area are presented in Table A1.1. According to the most recent population census, the population size of the RMM project area was 36,661 in 2010.[1] Based on the official population growth estimates for the Northern region of 2.9% per annum, the projected population size of the RMM project area in 2014 is approximately 41,102.

**Figure A.1. Map of Ghana showing the Northern Region and the three districts selected for the RRT project**

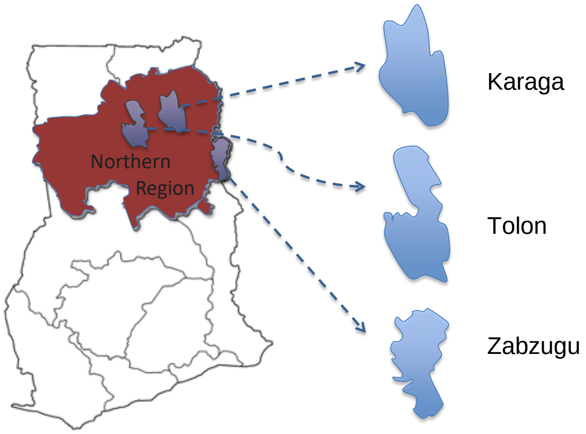


[1] Ghana Statistical Service, Government of Ghana. 2010 Population and Housing Census: Final Results. Available: http://www.statsghana.gov.gh/docfiles/2010phc/2010_POPULATION_AND_HOUSING_CENSUS_FINAL_RESULTS.pdf. Accessed January 4, 2016.

**Table A1.1. Demographic characteristics for Ghana, the Northern Region, and RMM project areas**

| Indicator | Value | Source |
|---|---|---|
| **Population** | | |
| National | 24,658,823 | 2010 Census |
| Northern Region | 2,479,461 | 2010 Census |
| Karaga District | 77,706 | 2010 Census |
| Tolon District | 112,331 | 2010 Census |
| Zabzugu District | 123,854 | 2010 Census |
| RMM project area | 36,661 | 2010 Census |
| **Under-5 population (% of total pop)** | | |
| National | 3,405,406 (13.8%) | 2010 Census |
| Northern Region | 423,024 (17.1%) | 2010 Census |
| **Total fertility rate** | | |
| National | 4.3 | 2011 MICS |
| Northern Region | 6.2* | 2011 MICS |
| **Under-5 mortality rate** | | |
| National | 82 per 1000 | 2011 MICS |
| Northern Region | 124 per 1000* | 2011 MICS |

*Highest regional rate in the country

The RMM community-based method relied on the community as the primary functioning work unit for the collection of birth and death information by the CBVs. We therefore elected to use the community as the basic selection unit in the sample design. The options for a sampling frame for RMM in each district were: (1) a complete community list from the local district assembly, district health directorate, or other district level ministry office; or (2) an area frame based on the Enumeration Areas (EAs) of the Ghana 2010 Population and Household census. Due to our inability to confirm completeness of any list, and observed variations between the community lists gathered from the various district-level offices within each respective district, we opted for the area frame approach. We used the list of EAs from the Ghana 2010 Census as a base from which to select the communities and carry out the sampling. Although EAs are area-based units with geographical boundaries, they also correspond well with community boundaries.

For operational reasons, we used a “cluster” as the basic working unit of the RMM project. This cluster is the same as a community for most clusters. It is different for only a few larger communities, where the collection of data on births and under-five deaths requires several CBVs. For these larger communities, we defined the cluster as equivalent to an EA. No more than four EAs comprise a community. We assigned a three-digit number to each cluster, where the first digit identified the district, the second digit indicated the community size in three categories, and the third digit was an ordinal measure within the respective category. This cluster number acted as a key identifier during both fieldwork and data processing.

In May 2011, we selected 96 clusters (corresponding to 83 communities in 61 EAs) from three strata, using sampling proportional to population size in the three districts. We therefore used a stratified sampling strategy (in lieu of sampling EAs) to approximate communities as the

selection unit in the sample design for the RMM project. Because RMM was concerned with rural vital event registration, communities needed to be stratified by population size. The stratification introduced was based on the EA type code:

1 – EA with one locality
2 – More than one (and up to four) EAs forming one locality (i.e. larger communities)
3 – Two or more localities forming one EA (i.e. a cluster of smaller communities)

Table A1.2 provides a summary of the number of EAs per stratum for each of the districts, and shows the district samples by EA, community and cluster. Stratum 1 (EA type code = 1) equates one EA to one community and thus selecting an EA is equal to selecting a community.

**Table A1.2. Sub-division of Ghana RMM sample by district, EA, community and RMM cluster**

| | | Number of Units | | | Census 2010 | |
|---|---|---|---|---|---|---|
| **District** | **Stratum** | **EAs** | **Communities** | **RMM Clusters** | **Population*** | **Est. no. hhs**** |
| Karaga | 1 | 5 | 5 | 5 | | |
| | 2 | 8 | 3 | 8 | | |
| | 3 | 7 | 23 | 23 | | |
| | **Subtotal** | **20** | **31** | **36** | 11,617 | 2,003 |
| Tolon | 1 | 6 | 6 | 6 | | |
| | 2 | 8 | 3 | 8 | | |
| | 3 | 6 | 14 | 14 | | |
| | **Subtotal** | **20** | **23** | **28** | 11,774 | 2,003 |
| Zabzugu | 1 | 6 | 6 | 6 | | |
| | 2 | 5 | 2 | 5 | | |
| | 3 | 10 | 21 | 21 | | |
| | **Subtotal** | **21** | **29** | **32** | 13,270 | 2,288 |
| **TOTAL** | | **61** | **83** | **96** | **36,661** | **6,294** |

*The population figures come from the 2010 census.

**The household figures come from the conversion of population into households by using an average number of persons per household of 5.8, which was derived from the 2007 Multiple Indicator Cluster Survey using the Northern Region data.
