# Supplementary material for: Using community-based reporting of vital events to monitor child mortality: Lessons from rural Ghana
Source: PLoS One. 2018 Jan 30;13(1):e0192034. doi: 10.1371/journal.pone.0192034 (PMC5790256; doi:10.1371/journal.pone.0192034)
Supplement: S2 File — (PDF) [file pone.0192034.s002.pdf]

Supporting Information File 2: Sample monthly summary form (MSF)

BDR+ MONTHLY SUMMARY FORM

Month of report: ___________ Year ___________ Name of district ______________________________ Code: _________

Name of village __________________ Code: __________ Name of Community Volunteer: ___________________ Code: _________

Date of collection by supervisor ____/____/______ Supervisor signature _______________________________________

RTHS							
other of Child	Father of Child	Child					
me	Name	Name	BDR+ Code	Date of birth (DD/MM/YYYY)	Sex (M/F)	Place of Birth (1=home; 2=facility; 3=other)	Attendant (Yes/No)
			___/___/__	__/__/____			
			___/___/__	__/__/____			
			___/___/__	__/__/____			
			___/___/__	__/__/____			
			___/___/__	__/__/____			
			___/___/__	__/__/____			

ATHS								COMMENTS
me	BDR+ Code	Date of birth (DD/MM/YYYY)	Age at death (yrs)	Date of death (DD/MM/YYYY)	Sex (M/F)	Reported cause of death	Place of death (1=home; 2=facility; 3=Other)	
	___/___/__	__/__/____		__/__/____				
	___/___/__	__/__/____		__/__/____				
	___/___/__	__/__/____		__/__/____				

PREGNANCIES						
No.	Name of pregnant Woman	BDR+ Code	Number of Months Pregnant	Pregnancy outcome / result (1=live birth; 2=still birth; 3=other)	Date of Outcome (Birth or Other) (DD/MM/YYYY)	Comment
1.		___/___/__			__/__/____ (DD /MM / Y Y Y Y)	
		___/___/__			__/__/____	
		___/___/__			__/__/____	
		___/___/__			__/__/____	
		___/___/__			__/__/____	
		___/___/__			__/__/____	
		___/___/__			__/__/____	
		___/___/__			__/__/____	
		___/___/__			__/__/____	
		___/___/__			__/__/____	
