# Supplementary material for: Using community-based reporting of vital events to monitor child mortality: Lessons from rural Ghana
Source: PLoS One. 2018 Jan 30;13(1):e0192034. doi: 10.1371/journal.pone.0192034 (PMC5790256; doi:10.1371/journal.pone.0192034)
Supplement: S3 File — (PDF) [file pone.0192034.s003.pdf]

# Supporting Information File 3: Study questionnaires (end-of-project census)

[Questionnaires begin on next page]

**ELS2**

# HOUSEHOLD QUESTIONNAIRE

| HOUSEHOLD INFORMATION PANEL | HH |
|---|---|
| HH1A. BDR+ Cluster number: ___ ___ ___<br>HH1B. Community name: ______________________ | HH2. Household number: __ __ __ \| __ __ |
| HH3. Interviewer name and number:<br>Name _________________________ ___ ___ | HH4. Supervisor name and number:<br>Name__________________________ ___ ___ |
| HH5. Day / Month / Year of interview: ___ ___ / ___ ___ / ___ ___ ___ ___ | |
| HH6. Area:<br>Urban ........................................................ 1<br>Rural.......................................................... 2 | HH7. District:<br>ZabzuguTatali ............................................... 1<br>Tolon Kumbungu.......................................... 2<br>Karaga......................................................... 3 |

*Read the household consent form for the Household Questionnaire respondent before going further in the questionnaire.*

*Is permission given?*

☐ *Yes, permission is given* ⇨ *Go to HH17 (on next page) to record the time and then begin the interview.*

☐ *No, permission is not given* ⇨ *Complete HH9. Discuss this result with your supervisor.*

| *After all questionnaires for the household have been completed, fill in the following information:* | |
|---|---|
| HH8. Name of head of household: | ___________________________________________ |
| HH9. Result of household interview:<br>Completed ........................................................01<br>No household member or no competent respondent at home at time of visit ....... 02<br>Entire household absent for extended period of time......................................... 03<br>Refused .......................................................... 04<br>Dwelling vacant / Address not a dwelling ....... 05<br>Dwelling destroyed ......................................... 06<br>Dwelling not found .......................................... 07<br>Other (*specify*) _______________________ 96 | HH10. Respondent to household questionnaire:<br>Name:___________________________________<br>Line Number: ___ ___<br><br>HH11. Total number of household members: ___ ___ |
| HH12. Number of women age 15-49 years: ___ ___ | HH13. Number of woman's questionnaires completed: ___ ___ |
| HH14. Number of under-5s : ....................___ ___ | HH15. Number of birth registration questionnaires completed: ............ ___ ___ |

| *Interviewer notes* |
|---|
| |

| HH16. Field edited by (name and number): ______________________________________ ___ ___ |
|---|

| |
|---|
| HH17. *Record the time.* |
| Hours......... __ __ |
| Minutes...... __ __ |

## HOUSEHOLD LISTING FORM HL

FIRST, PLEASE TELL ME THE NAME OF EACH PERSON WHO USUALLY LIVES HERE, STARTING WITH THE HEAD OF THE HOUSEHOLD.

*List the head of the household in line 01. List all household members (HL2), their relationship to the household head (HL3), and their sex (HL4)*

*Then ask:* ARE THERE ANY OTHERS WHO LIVE HERE, EVEN IF THEY ARE NOT AT HOME NOW?

*If yes, complete listing for questions HL2-HL4. Then, ask questions starting with HL5 for each person at a time.*

| HL1. *Line no.* | HL2. *Name* | HL3. WHAT IS THE RELATION-SHIP OF (*name*) TO THE HEAD OF HOUSE-HOLD? | HL4. IS (*name*) MALE OR FEMALE? 1 Male 2 Female | HL5. WHAT IS (*name*)'s DATE OF BIRTH? 98 DK 9998 DK *Ensure that every person less than 6 years of age has a month and year of birth* | | HL6. HOW OLD IS (*name*)? *Record in completed years. If age is 95 or above, record '95'* | *For household members age **15 and above*** HL7. WHAT IS (*name*)'s CURRENT MARITAL STATUS? 1 Married or living together 2 Divorced/ separated 3 Widowed 4 Never married or lived together | *Women age **15-49*** HL8. *Circle line number if woman is age **15-49*** | *For children **under 5 years** of age* HL9 *Circle line number if child is **under 5 years** of age* | HL10. WHO IS THE MOTHER OR PRIMARY CARETAKER OF THIS CHILD? *Record line number of mother/ caretaker* | *For all household members* HL11. WAS (*name*) BORN AT HOME, IN A HEALTH FACILITY OR OTHER PLACE? 1 Home 2 Health facility 3. Other 8. DK | HL12. WHAT IS (*name*)'s NATIONALITY? *Insert appropriate nationality code* | HL13. WHERE WAS (*name*) BORN? IN THIS VILLAGE OR TOWN? | ELSEWHERE? *Insert village or town name and appropriate birthplace code* | | HL14. FOR HOW MANY YEARS HAS (*name*) LIVED IN THIS VILLAGE/ TOWN? *Record zero if less than one year* |
|---|---|---|---|---|---|---|---|---|---|---|---|---|---|---|---|---|
| Line | Name | Relation | M F | MM | Year | Age | Marital status | 15-49 | Under 5 | Mother | Place | Nationality | Y N | Village/Town | Code | Years |
| 01 | | 0 1 | 1 2 | _ _ | _ _ _ _ | __ __ | 1 2 3 4 | 01 | 01 | __ __ | 1 2 3 8 | __ __ | 1 2→ | ____________ | __ __ | __ __ |
| 02 | | __ __ | 1 2 | _ _ | _ _ _ _ | __ __ | 1 2 3 4 | 02 | 02 | __ __ | 1 2 3 8 | __ __ | 1 2→ | ____________ | __ __ | __ __ |
| 03 | | __ __ | 1 2 | _ _ | _ _ _ _ | __ __ | 1 2 3 4 | 03 | 03 | __ __ | 1 2 3 8 | __ __ | 1 2→ | ____________ | __ __ | __ __ |
| 04 | | __ __ | 1 2 | _ _ | _ _ _ _ | __ __ | 1 2 3 4 | 04 | 04 | __ __ | 1 2 3 8 | __ __ | 1 2→ | ____________ | __ __ | __ __ |
| 05 | | __ __ | 1 2 | _ _ | _ _ _ _ | __ __ | 1 2 3 4 | 05 | 05 | __ __ | 1 2 3 8 | __ __ | 1 2→ | ____________ | __ __ | __ __ |
| 06 | | __ __ | 1 2 | _ _ | _ _ _ _ | __ __ | 1 2 3 4 | 06 | 06 | __ __ | 1 2 3 8 | __ __ | 1 2→ | ____________ | __ __ | __ __ |
| 07 | | __ __ | 1 2 | _ _ | _ _ _ _ | __ __ | 1 2 3 4 | 07 | 07 | __ __ | 1 2 3 8 | __ __ | 1 2→ | ____________ | __ __ | __ __ |
| 08 | | __ __ | 1 2 | _ _ | _ _ _ _ | __ __ | 1 2 3 4 | 08 | 08 | __ __ | 1 2 3 8 | __ __ | 1 2→ | ____________ | __ __ | __ __ |
| 09 | | __ __ | 1 2 | _ _ | _ _ _ _ | __ __ | 1 2 3 4 | 09 | 09 | __ __ | 1 2 3 8 | __ __ | 1 2→ | ____________ | __ __ | __ __ |
| 10 | | __ __ | 1 2 | _ _ | _ _ _ _ | __ __ | 1 2 3 4 | 10 | 10 | __ __ | 1 2 3 8 | __ __ | 1 2→ | ____________ | __ __ | __ __ |

| HL1. Line no. | HL2. Name | HL3. WHAT IS THE RELATIONSHIP OF (name) TO THE HEAD OF HOUSEHOLD? | HL4. IS (name) MALE OR FEMALE? 1 Male 2 Female | HL5. WHAT IS (name)'s DATE OF BIRTH? 98 DK 9998 DK *Ensure that every person less than 6 years of age has a month and year of birth* | | HL6. HOW OLD IS (name)? *Record in completed years. If age is 95 or above, record '95'* | HL7. WHAT IS (name)'s CURRENT MARITAL STATUS? 1 Married or living together 2 Divorced/ separated 3 Widowed 4 Never married or lived together | HL8. *Circle line number if woman is age* **15-49** | HL9 *Circle line number if child is* **under 5 years** *of age* | HL10. WHO IS THE MOTHER OR PRIMARY CARETAKER OF THIS CHILD? *Record line number of mother/ caretaker* | HL11. WAS (name) BORN AT HOME, IN A HEALTH FACILITY OR OTHER PLACE? 1 Home 2 Health facility 3. Other 8. DK | HL12. WHAT IS (name)'s NATIONALITY? *Insert appropriate nationality code* | HL13. WHERE WAS (name) BORN? IN THIS VILLAGE OR TOWN? | ELSEWHERE? *Insert village or town name and appropriate birthplace code* | | HL14. FOR HOW MANY YEARS HAS (name) LIVED IN THIS VILLAGE/ TOWN? *Record zero if less than one year* |
|---|---|---|---|---|---|---|---|---|---|---|---|---|---|---|---|---|
| Line | Name | Relation | M F | MM | Year | Age | Marital status | 15-49 | Under 5 | Mother | Place | Nationality | Y N | Village/Town | Code | Years |
| 11 | | __ __ | 1 2 | _ _ | _ _ _ _ | __ __ | 1 2 3 4 | 11 | 11 | __ __ | 1 2 3 8 | __ __ | 1 2→ | ________ | __ __ | __ __ |
| 12 | | __ __ | 1 2 | _ _ | _ _ _ _ | __ __ | 1 2 3 4 | 12 | 12 | __ __ | 1 2 3 8 | __ __ | 1 2→ | ________ | __ __ | __ __ |
| 13 | | __ __ | 1 2 | _ _ | _ _ _ _ | __ __ | 1 2 3 4 | 13 | 13 | __ __ | 1 2 3 8 | __ __ | 1 2→ | ________ | __ __ | __ __ |
| 14 | | __ __ | 1 2 | _ _ | _ _ _ _ | __ __ | 1 2 3 4 | 14 | 14 | __ __ | 1 2 3 8 | __ __ | 1 2→ | ________ | __ __ | __ __ |
| 15 | | __ __ | 1 2 | _ _ | _ _ _ _ | __ __ | 1 2 3 4 | 15 | 15 | __ __ | 1 2 3 8 | __ __ | 1 2→ | ________ | __ __ | __ __ |

*Tick here if additional questionnaire used* ☐

*Probe for additional household members.*
*Probe especially for any infants or small children not listed, and others who may not be members of the family (such as servants, friends) but who usually live in the household.*

*Now for each woman age 15-49 years, write her name and line number and other identifying information in the information panel of a separate Women's Questionnaire.*
*You should now have a separate questionnaire for each eligible woman in the household.*

** Codes for HL3: Relationship to head of household: ....*

01 Head
02 Wife / Husband
03 Son / Daughter
04 Son-In-Law / Daughter-In-Law
05 Grandchild
06 Parent
07 Parent-In-Law
08 Brother / Sister
09 Brother-In-Law / Sister-In-Law
10 Uncle / Aunt
11 Niece / Nephew
12 Other relative
13 Adopted / Foster / Stepchild
14 Not related
98 Don't know

*Codes for HL12: Nationality*

01 Ghanaian by birth
02 Ghanaian by naturalisation
03 Dual nationality (Ghanaian & other)
04 African
05 European
06 American (North, South & Caribbean
07 Asian & Oceanian (incl. Australia & New Zealand)
98 Don't know

*Codes for HL13: Birthplace*

01 Western region
02 Central region
03 Greater Accra
04 Volta region
05 Eastern region
06 Ashanti region
07 Brong Ahafo region
08 Northern region
09 Upper East
10 Upper West
11 Africa
12 Europe
13 Americas (North, South & Caribbean)
14 Asia
15 Oceania
98 Don't know

## RESIDENCE AND EDUCATION ED

| | | | *For all household members* | | | *For those **11years of age and above*** | *For those **5 years of age and above*** | | | | | |
|---|---|---|---|---|---|---|---|---|---|---|---|---|
| ED1.<br>*Line no.* | ED2.<br>*Name and age*<br>*Copy from Household Listing Form, HL2 and HL6* | | ED3.<br>DID (*name*) STAY HERE LAST NIGHT?<br>1 Yes<br>2 No | ED4.<br>DID (*name*) LIVE HERE MOST OF THE <u>LAST 6 MONTHS</u>?<br>1 Yes<br>2 No | ED5.<br>DOES (*name*) EXPECT TO LIVE HERE FOR MOST OF THE <u>NEXT 6 MONTHS</u>?<br>**1 Yes**<br>**2 No**<br>**8 DK** | ED6.<br>IN WHAT LANGUAGE CAN (*name*) READ AND WRITE?<br>Code:<br>1 None (not literate)<br>2 English only<br>3 Ghanian only<br>4 English & Ghanaian<br>5 English & French<br>6 Other language than Ghanaian, English or French<br>8 DK | ED7.<br>HAS (*name*) EVER ATTENDED SCHOOL OR PRE-SCHOOL?<br>1 Yes<br>2 No ⇘ *Next line* | ED8.<br>DURING THE PRESENT SCHOOL YEAR, DID (*name*) ATTEND SCHOOL OR PRESCHOOL AT ANY TIME?<br>1 Yes ⇨ED10<br>2 No | ED9.<br>WHAT IS THE HIGHEST LEVEL OF SCHOOL (*name*) ATTENDED?<br>WHAT IS HIGHEST GRADE (*name*) COMPLETED AT THIS LEVEL?<br>Level:<br>0 Preschool<br>1 Primary<br>2 Junior HS<br>3 Senior HS<br>4 Higher<br>8 DK<br>*If level=0, skip to next line* | Grade:<br>98 DK<br>*If less than 1 grade, enter 00.* | ED10.<br>DURING THIS SCHOOL YEAR, WHICH LEVEL AND GRADE IS (*name*) ATTENDING?<br>Level:<br>0 Preschool<br>1 Primary<br>2 Junior HS<br>3 Senior HS<br>4 Higher<br>8 DK<br>*If level=0, skip to next line* | Grade<br>98 DK |
| Line | Name | Age | Yes No | Yes No | Yes No DK | Literacy code | Yes No | Yes No | Level | Grade | Level | Grade |
| 01 | | __ __ | 1 2 | 1 2 | 1 2 8 | 1 2 3 4 5 6 8 | 1 2 | 1⇨ED10 2 | 0 1 2 3 4 8 | __ __ *Next line* | 0 1 2 3 4 8 | __ __ *Next line* |
| 02 | | __ __ | 1 2 | 1 2 | 1 2 8 | 1 2 3 4 5 6 8 | 1 2 | 1⇨ED10 2 | 0 1 2 3 4 8 | __ __ *Next line* | 0 1 2 3 4 8 | __ __ *Next line* |
| 03 | | __ __ | 1 2 | 1 2 | 1 2 8 | 1 2 3 4 5 6 8 | 1 2 | 1⇨ED10 2 | 0 1 2 3 4 8 | __ __ *Next line* | 0 1 2 3 4 8 | __ __ *Next line* |
| 04 | | __ __ | 1 2 | 1 2 | 1 2 8 | 1 2 3 4 5 6 8 | 1 2 | 1⇨ED10 2 | 0 1 2 3 4 8 | __ __ *Next line* | 0 1 2 3 4 8 | __ __ *Next line* |
| 05 | | __ __ | 1 2 | 1 2 | 1 2 8 | 1 2 3 4 5 6 8 | 1 2 | 1⇨ED10 2 | 0 1 2 3 4 8 | __ __ *Next line* | 0 1 2 3 4 8 | __ __ *Next line* |
| 06 | | __ __ | 1 2 | 1 2 | 1 2 8 | 1 2 3 4 5 6 8 | 1 2 | 1⇨ED10 2 | 0 1 2 3 4 8 | __ __ *Next line* | 0 1 2 3 4 8 | __ __ *Next line* |
| 07 | | __ __ | 1 2 | 1 2 | 1 2 8 | 1 2 3 4 5 6 8 | 1 2 | 1⇨ED10 2 | 0 1 2 3 4 8 | __ __ *Next line* | 0 1 2 3 4 8 | __ __ *Next line* |
| 08 | | __ __ | 1 2 | 1 2 | 1 2 8 | 1 2 3 4 5 6 8 | 1 2 | 1⇨ED10 2 | 0 1 2 3 4 8 | __ __ *Next line* | 0 1 2 3 4 8 | __ __ *Next line* |
| 09 | | __ __ | 1 2 | 1 2 | 1 2 8 | 1 2 3 4 5 6 8 | 1 2 | 1⇨ED10 2 | 0 1 2 3 4 8 | __ __ *Next line* | 0 1 2 3 4 8 | __ __ *Next line* |
| 10 | | __ __ | 1 2 | 1 2 | 1 2 8 | 1 2 3 4 5 6 8 | 1 2 | 1⇨ED10 2 | 0 1 2 3 4 8 | __ __ *Next line* | 0 1 2 3 4 8 | __ __ *Next line* |
| 11 | | __ __ | 1 2 | 1 2 | 1 2 8 | 1 2 3 4 5 6 8 | 1 2 | 1⇨ED10 2 | 0 1 2 3 4 8 | __ __ *Next line* | 0 1 2 3 4 8 | __ __ *Next line* |
| 12 | | __ __ | 1 2 | 1 2 | 1 2 8 | 1 2 3 4 5 6 8 | 1 2 | 1⇨ED10 2 | 0 1 2 3 4 8 | __ __ *Next line* | 0 1 2 3 4 8 | __ __ *Next line* |
| 13 | | __ __ | 1 2 | 1 2 | 1 2 8 | 1 2 3 4 5 6 8 | 1 2 | 1⇨ED10 2 | 0 1 2 3 4 8 | __ __ *Next line* | 0 1 2 3 4 8 | __ __ *Next line* |
| 14 | | __ __ | 1 2 | 1 2 | 1 2 8 | 1 2 3 4 5 6 8 | 1 2 | 1⇨ED10 2 | 0 1 2 3 4 8 | __ __ *Next line* | 0 1 2 3 4 8 | __ __ *Next line* |
| 15 | | __ __ | 1 2 | 1 2 | 1 2 8 | 1 2 3 4 5 6 8 | 1 2 | 1⇨ED10 2 | 0 1 2 3 4 8 | __ __ *Next line* | 0 1 2 3 4 8 | __ __ *Next line* |

## MIGRATION MI

THE FOLLOWING ARE QUESTIONS ABOUT ANY REGULAR MEMBERS OF THE HOUSEHOLD THAT MAY CURRENTLY BE AWAY FOR MIGRANT WORK PURPOSES

| | | |
|---|---|---|
| MI1. ARE THERE ANY OTHER MEMBERS OF THE HOUSEHOLD THAT USUALLY LIVE HERE DURING OTHER PERIODS OF THE YEAR THAT YOU DIDN'T MENTION BEFORE? | Yes.... ..1<br>No........ 2 | 2⇨BR1 |

MI2. PLEASE TELL ME A LITTLE ABOUT ALL MEMBERS WHO ARE AWAY.

| MI3. *Line no.* *Continue from last HL1 no* | MI4. *Name* | MI5. HOW OLD IS (*name*)? *Insert '95' if aged 95 or older* | MI6. WHAT IS THE RELATION-SHIP OF (*name*) TO THE HEAD OF HOUSE-HOLD? | MI7. IS (*name*) MALE OR FEMALE? 1 Male 2 Female | MI8. WHEN DID (*name*) LEAVE THIS COMMUNITY? *Must put a month and year for all persons entered here* | | MI9. WHERE DID (*name*) GO? 1 Accra 2 Kumasi 3 Tamale 6 Other (*specify*) | MI10. WHAT DID (*name*) GO TO DO? 1 Farm laborer 2 Kayayo 3 Collect scrap metals 6 Other (*specify*) | M11. WHEN DO YOU EXPECT (*name*) TO RETURN TO THIS COMMUNITY? | |
|---|---|---|---|---|---|---|---|---|---|---|
| Line | Name | | Relation | M F | MM | Year | Location | Activity | MM | Year |
| __ __ | | __ __ | __ __ | 1 2 | __ __ | __ __ __ __ | 1 2 3<br>6 → ____________ | 1 2 3<br>6 → ____________ | __ __ | __ __ __ __ |
| __ __ | | __ __ | __ __ | 1 2 | __ __ | __ __ __ __ | 1 2 3<br>6 → ____________ | 1 2 3<br>6 → ____________ | __ __ | __ __ __ __ |
| __ __ | | __ __ | __ __ | 1 2 | __ __ | __ __ __ __ | 1 2 3<br>6 → ____________ | 1 2 3<br>6 → ____________ | __ __ | __ __ __ __ |
| __ __ | | __ __ | __ __ | 1 2 | __ __ | __ __ __ __ | 1 2 3<br>6 → ____________ | 1 2 3<br>6 → ____________ | __ __ | __ __ __ __ |
| __ __ | | __ __ | __ __ | 1 2 | __ __ | __ __ __ __ | 1 2 3<br>6 ____________ | 1 2 3<br>6 → ____________ | __ __ | __ __ __ __ |
| __ __ | | __ __ | __ __ | 1 2 | __ __ | __ __ __ __ | 1 2 3<br>6 → ____________ | 1 2 3<br>6 → ____________ | __ __ | __ __ __ __ |

*Tick here if additional questionnaire used* ☐

*Codes for MI6: Relationship to head of household:*

| | | | |
|---|---|---|---|
| 01 Head<br>02 Wife / Husband<br>03 Son / Daughter<br>04 Son-In-Law / Daughter-In-Law | 05 Grandchild<br>06 Parent<br>07 Parent-In-Law<br>08 Brother / Sister | 09 Brother-In-Law / Sister-In-Law<br>10 Uncle / Aunt<br>11 Niece / Nephew<br>12 Other relative | 13 Adopted / Foster / Stepchild<br>14 Not related<br>98 Don't know |

| BIRTH REGISTRATION | | | BR |
|---|---|---|---|
| THE FOLLOWING ARE QUESTIONS ABOUT THE REGISTRATION OF A CHILD'S BIRTH. | | | |
| BR1. DO YOU KNOW WHERE TO REGISTER A CHILD'S BIRTH? | Yes ........ 1<br>No ........ 2 | | 2⇨BR3 |
| BR2. CAN YOU TELL ME WHERE YOU CAN REGISTER A CHILD'S BIRTH? IS IT AT A BDR OFFICE, WITH A COMMUNITY BASED VOLUNTEER (CBV), AT A MASS REGISTRATION DURING HEALTH PROMOTION WEEK, OR SOMEWHERE ELSE?<br>*Probe:* ANYWHERE OR ANYONE ELSE?<br>*Probe for location or type of person and circle all answers given.* | BDR Office ........ A<br>CBV ........ B<br>Mass registration ........ C<br>Other (*Specify*) ________ X | | |
| BR3. I NOW HAVE A FEW QUESTIONS ABOUT EACH CHILD UNDER AGE 5 YEARS IN YOUR HOUSEHOLD. | | | |

| BR4.<br>*Line no. from HL1* | BR5.<br>*Name, sex and age*<br>*Copy from Household Listing Form, HL2 HL4 and HL6*<br>*List only those children who are less than 5 years of age* | | | BR6.<br>WHAT IS YOUR RELATIONSHIP TO (*name*)?<br>1 Father<br>2 Mother<br>6 Other (*Specify*) | BR7.<br>HAS (*name*)'S BIRTH BEEN REGISTERED WITH THE BIRTHS AND DEATHS REGISTRY?<br>1 Yes → BR8<br>2 NO → BR9<br>8 DK → WHO WOULD KNOW? *Ask for person line no.* | BR8.<br>WHO REGISTERED (*name*)'S BIRTH?<br>1 Respondent<br>2 Other (*enter line no.*)<br>8 DK<br>*Then go to next child* | BR9.<br>WHY WAS (*name*)'S BIRTH NOT REGISTERED?<br>1 Costs too much<br>2 Must travel too far<br>3 Did not know the birth should be registered<br>4 Did not want to pay fine<br>5 Did not know where to register<br>6 Other (*specify*)<br>8 DK<br>*Then go to next child* |
|---|---|---|---|---|---|---|---|
| Line | Name | Sex | Age | Relationship | Yes No DK → *line no.* | Resp. Other (line no) DK | Reason |
| __ __ | | 1 2 | __ __ | 1 2<br>6 -> ________ | 1 2 8 → __ __ | 1 2 → __ __ 8 | 1 2 3 4 5<br>6 -> ________ 8 |
| __ __ | | 1 2 | __ __ | 1 2<br>6 -> ________ | 1 2 8 → __ __ | 1 2 → __ __ 8 | 1 2 3 4 5<br>6 -> ________ 8 |
| __ __ | | 1 2 | __ __ | 1 2<br>6 -> ________ | 1 2 8 → __ __ | 1 2 → __ __ 8 | 1 2 3 4 5<br>6 -> ________ 8 |
| __ __ | | 1 2 | __ __ | 1 2<br>6 -> ________ | 1 2 8 → __ __ | 1 2 → __ __ 8 | 1 2 3 4 5<br>6 -> ________ 8 |
| __ __ | | 1 2 | __ __ | 1 2<br>6 -> ________ | 1 2 8 → __ __ | 1 2 → __ __ 8 | 1 2 3 4 5<br>6 -> ________ 8 |
| __ __ | | 1 2 | __ __ | 1 2<br>6 -> ________ | 1 2 8 → __ __ | 1 2 → __ __ 8 | 1 2 3 4 5<br>6 -> ________ 8 |

*Tick here if additional questionnaire used* ☐

| REGISTRATION OF A DEATH | | DR |
|---|---|---|
| AS WELL AS REGISTRATION OF BIRTH, REGISTRATION OF DEATH IS AN IMPORTANT COMMUNITY RESPONSIBILITY. | | |
| DR1. IF A PERSON DIES IN THIS COMMUNITY, DOES AN AUTHORIZATION FOR BURIAL HAVE TO BE OBTAINED? | Yes ... 1<br>No ... 2<br>DK ... 8 | <br>2⇨DR3<br>8⇨DR3 |
| DR2. FROM WHOM CAN THIS AUTHORIZATION BE OBTAINED?<br>*Probe:*<br>ANYONE ELSE?<br>*Probe for the type of person and circle all answers given.* | Chief of community ... A<br>Imam ... B<br>Priest ... C<br>CBV ... D<br>Nurse or Midwife ... E<br>Other (*specify*) ______ X | |
| DR3. IN THE LAST FIVE YEARS, HAS A CHILD THAT WAS LESS THAN 5 YEARS OF AGE DIED IN THIS HOUSEHOLD? | Yes ... 1<br>No ... 2 | <br>2⇨HH18 |
| DR4. WAS AN AUTHORIZATION FOR BURIAL OBTAINED FOR THIS CHILD? | Yes ... 1<br>No ... 2<br>DK ... 8 | <br>2⇨DR7<br>8⇨DR7 |
| DR5. FROM WHOM WAS THIS AUTHORIZATION OBTAINED? | Chief of community ... 1<br>Imam ... 2<br>Priest ... 3<br>CBV ... 4<br>Nurse or Midwife ... 5<br>Other (*specify*) ______ 6 | |
| DR6. WAS THE DEATH OF THIS CHILD REPORTED TO ANYONE ELSE OF IMPORTANCE IN THE COMMUNITY, SUCH AS THE CHIEF OR THE IMAM? | Yes ... 1<br>No ... 2<br>DK ... 8 | 1⇨DR8<br>2⇨HH18<br>8⇨HH18 |
| DR7. WAS THE DEATH REPORTED TO SOMEONE OF IMPORTANCE IN THE COMMUNITY, SUCH AS THE CHIEF, THE IMAM OR THE PRIEST? | Yes ... 1<br>No ... 2<br>DK ... 8 | <br>2⇨HH18<br>8⇨HH18 |
| DR8. WHO WAS THIS PERSON?<br>*Probe:*<br>ANYONE ELSE?<br>*Probe for the type of person and circle all answers given.* | Chief of community ... A<br>Imam ... B<br>Priest ... C<br>CBV ... D<br>Nurse or Midwife ... E<br>Other (*specify*) ______ X | |

| WATER AND SANITATION | | WS |
|---|---|---|
| WS1. WHAT IS THE <u>MAIN</u> SOURCE OF DRINKING WATER FOR MEMBERS OF YOUR HOUSEHOLD? | Piped water<br>Piped into dwelling ................................ 11<br>Piped into compound, yard or plot ........ 12<br>Piped to neighbour ................................ 13<br>Public tap / standpipe ............................ 14<br>Tube Well, Borehole ................................ 21<br>Dug well<br>Protected well........................................ 31<br>Unprotected well .................................. 32<br>Water from spring<br>Protected spring .................................... 41<br>Unprotected spring................................ 42<br>Rainwater collection ................................. 51<br>Tanker-truck ............................................. 61<br>Cart with small tank / drum ....................... 71<br>Surface water (river, stream, dam, lake, pond, canal, irrigation channel)............. 81<br><br>Bottled water ............................................ 91<br><br>Other (*specify*)_____________________ 96 | |
| WS2. WHAT KIND OF TOILET FACILITY DO MEMBERS OF YOUR HOUSEHOLD USUALLY USE?<br><br>*If "flush" or "pour flush", probe:*<br>WHERE DOES IT FLUSH TO?<br><br>*If necessary, ask permission to observe the facility.* | Flush / Pour flush<br>Flush to piped sewer system ................ 11<br>Flush to septic tank ............................... 12<br>Flush to pit (latrine) ............................... 13<br>Flush to somewhere else ...................... 14<br>Flush to unknown place / Not sure / DK where.......................................... 15<br>Pit latrine<br>Ventilated Improved Pit latrine (VIP) .... 21<br>Pit latrine with slab ................................ 22<br>Pit latrine without slab / Open pit .......... 23<br><br>Composting toilet ...................................... 31<br>Bucket ....................................................... 41<br>Hanging toilet, hanging latrine .................. 51<br><br>No facility, bush, field ................................ 95<br><br>Other (*specify*) _____________________ 96 | 95⇨Next Module |
| WS3. DO YOU SHARE THIS FACILITY WITH OTHERS WHO ARE NOT MEMBERS OF YOUR HOUSEHOLD? | Yes ............................................................. 1<br>No................................................................ 2 | 2⇨Next Module |
| WS4. HOW MANY HOUSEHOLDS IN TOTAL USE THIS TOILET FACILITY, INCLUDING YOUR OWN HOUSEHOLD? | Number of households (if less than 10) .. 0__<br><br>Ten or more households ........................... 10<br><br>DK ............................................................ 98 | |

| HOUSEHOLD CHARACTERISTICS | | HC |
|---|---|---|
| HC1A. WHAT IS THE RELIGION OF THE HEAD OF THIS HOUSEHOLD? | Catholic 11<br>Protestant (Anglican, Lutheran, Presbyterian, Methodist, etc.) 12<br>Pentecostal/Charismatic 13<br>Other Christian 14<br>Moslem 21<br>Traditional/Spiritualist 31<br>Other (*specify*) ______ 96<br>No religion 41 | |
| HC1B. TO WHAT ETHNIC GROUP DOES THE HEAD OF THIS HOUSEHOLD BELONG? | Basari 11<br>Ewe 12<br>Fulani 21<br>Gruma 31<br>Kokomba 32<br>Mole-Dagbani 41<br>Other (*specify*) ______ 96 | |
| HC1C. IN THE LAST 4 WEEKS DID THE HEAD OF THIS HOUSEHOLD DO ANY ACTIVITY OF AT LEAST ONE HOUR FOR PAY OR PROFIT OR FAMILY GAIN (IN CASH OR IN KIND)? | Yes.......... 1<br>No .......... 2 | 1⇨HC1E<br>2⇨HC1D |
| HC1D. WHAT WAS THE HEAD OF HOUSEHOLD DOING? | Did not work but had job to go back to (seasonal farmer, but currently no farming activities) 1<br>Did not work, but had job to go back to (other than farming) 2<br>Unemployed, worked before and available to work now 3<br>Unemployed, never worked before 4<br>Did not work before and not seeking work now 5<br>Disabled 6<br>Retired 7<br>DK 8 | 1⇨HC2<br>2⇨HC1E<br>3⇨HC1E<br>4–8 ⇨HC2 |
| HC1E. WHAT KIND OF WORK DID THE HEAD OF HOUSEHOLD DO?<br>*This refers to the type of work the head of household was doing. Use two or more words. For example, street trader, cattle farmer, primary schoolteacher, domestic worker, truck driver, filing clerk, etc.* | Description of work:<br>----------.<br>----------.<br>----------. | |
| HC2. HOW MANY ROOMS IN THIS HOUSEHOLD ARE USED FOR SLEEPING? | Number of rooms .......... __ __ | |
| HC3. *Main material of the dwelling floor.*<br>*Record observation.* | Natural floor<br>Earth / Sand .......... 11<br>Dung .......... 12<br>Rudimentary floor<br>Wood planks .......... 21<br>Palm / Bamboo .......... 22<br>Finished floor<br>Parquet or polished wood .......... 31<br>Vinyl or asphalt strips.......... 32<br>Ceramic tiles .......... 33<br>Cement .......... 34<br>Carpet .......... 35<br>Other (*specify*) ______ 96 | |

| | | |
|---|---|---|
| HC4. *Main material of the roof.*<br><br>*Record observation.* | Natural roofing<br>No Roof ........ 11<br>Thatch / Palm leaf ........ 12<br>Sod ........ 13<br>Rudimentary Roofing<br>Rustic mat ........ 21<br>Palm / Bamboo ........ 22<br>Wood planks ........ 23<br>Cardboard ........ 24<br>Finished roofing<br>Metal ........ 31<br>Wood ........ 32<br>Calamine / Cement fibre ........ 33<br>Ceramic tiles ........ 34<br>Cement ........ 35<br>Roofing shingles ........ 36<br><br>Other (*specify*) ______________ 96 | |
| HC5. *Main material of the exterior walls.*<br><br>*Record observation.* | Natural walls<br>No walls ........ 11<br>Cane / Palm / Trunks ........ 12<br>Dirt/gravel ........ 13<br>Rudimentary walls<br>Bamboo with mud ........ 21<br>Stone with mud ........ 22<br>Uncovered adobe ........ 23<br>Plywood ........ 24<br>Cardboard ........ 25<br>Reused wood ........ 26<br>Finished walls<br>Cement ........ 31<br>Stone with lime / cement ........ 32<br>Bricks ........ 33<br>Cement blocks ........ 34<br>Covered adobe ........ 35<br>Covered dirt/gravel ........ 36<br>Wood planks / shingles ........ 37<br><br>Other (*specify*) ______________ 96 | |
| HC6. WHAT TYPE OF FUEL DOES YOUR HOUSEHOLD MAINLY USE FOR COOKING? | Electricity ........ 01<br>Liquefied Petroleum Gas (LPG) ........ 02<br>Natural gas ........ 03<br>Biogas ........ 04<br>Kerosene ........ 05<br><br>Coal / Lignite ........ 06<br>Charcoal ........ 07<br>Wood ........ 08<br>Straw / Shrubs / Grass ........ 09<br>Animal dung ........ 10<br>Agricultural crop residue ........ 11<br><br>No food cooked in household ........ 95<br><br>Other (*specify*) ______________ 96 | 01⇨HC8<br>02⇨HC8<br>03⇨HC8<br>04⇨HC8<br><br>95⇨HC8 |

| | | |
|---|---|---|
| HC7. IS THE COOKING USUALLY DONE IN THE HOUSE, IN A SEPARATE BUILDING, OR OUTDOORS?<br><br>*If 'In the house', probe*: IS IT DONE IN A SEPARATE ROOM USED AS A KITCHEN? | In the house<br>In a separate room used as kitchen ....... 1<br>Elsewhere in the house .......................... 2<br>In a separate building ................................ 3<br>Outdoors<br>Within the compound .............................. 4<br>Outside the compound............................ 5<br>Other (*specify*) ______________________6 | |
| HC8. DOES YOUR HOUSEHOLD HAVE:<br><br>[A] ELECTRICITY?<br>[B] A RADIO?<br>[C] A TELEVISION?<br>[D] A NON-MOBILE TELEPHONE?<br>[E] A REFRIGERATOR?<br>[F] A GENERATOR<br>[G] A COMPUTER<br>[H] A SEWING MACHINE<br>[I] A BED<br>[J] A TABLE<br>[K] A CUPBOARD/CABINET | Yes No<br><br>Electricity ........................................ 1 2<br>Radio................................................ 1 2<br>Television........................................ 1 2<br>Non-mobile telephone...................... 1 2<br>Refrigerator...................................... 1 2<br>Generator........................................ 1 2<br>Computer ........................................ 1 2<br>Sewing machine .............................. 1 2<br>Bed.................................................. 1 2<br>Table ............................................... 1 2<br>Cupboard/Cabinet............................ 1 2 | |
| HC9. DOES ANY MEMBER OF YOUR HOUSEHOLD OWN:<br>[A] A WATCH?<br>[B] A MOBILE TELEPHONE?<br>[C] A BICYCLE?<br>[D] A MOTORCYCLE OR SCOOTER?<br>[E] AN ANIMAL-DRAWN CART?<br>[F] A MOTOR-KING?<br>[G] A CAR OR TRUCK?<br>[H] TRACTOR?<br>[I] A BOAT WITHOUT A MOTOR?<br>[J] A BOAT WITH A MOTOR | Yes No<br>Watch................................................1 2<br>Mobile telephone ..............................1 2<br>Bicycle...............................................1 2<br>Motorcycle / Scooter ........................1 2<br>Animal drawn-cart.............................1 2<br>Motor-King ........................................1 2<br>Car / Truck ........................................1 2<br>Tractor...............................................1 2<br>Boat without motor............................1 2<br>Boat with motor.................................1 2 | |
| HC10. DO YOU OR SOMEONE LIVING IN THIS HOUSEHOLD OWN THIS DWELLING?<br><br>*If "No", then ask:* DO YOU RENT THIS DWELLING FROM SOMEONE NOT LIVING IN THIS HOUSEHOLD?<br><br>*If "Rented from someone else", circle "2". For other responses, circle "6".* | Own........................................................... 1<br>Rent ........................................................... 2<br><br>Other (Not owned/rented) (*Specify*)<br><br>________________________________. 6 | |

| | | |
|---|---|---|
| HC11. DOES ANY MEMBER OF THIS HOUSEHOLD OWN ANY LAND THAT CAN BE USED FOR AGRICULTURE? | Yes ... 1<br>No ... 2 | 2⇨HC13 |
| HC12. HOW MANY HECTARES, ACRES OR POLES OF AGRICULTURAL LAND DO MEMBERS OF THIS HOUSEHOLD OWN?<br><br>*If less than 1, record "00". If 95 or more, record '95'. If unknown, record '98'.* | Hectares ... ___ ___<br>Acres ... ___ ___<br>Poles ... ___ ___<br><br>DK ... 98 | |
| HC13. DOES THIS HOUSEHOLD OWN ANY LIVESTOCK, HERDS, OTHER FARM ANIMALS, OR POULTRY? | Yes ... 1<br>No ... 2 | 2⇨HC15 |
| HC14. HOW MANY OF THE FOLLOWING ANIMALS DOES THIS HOUSEHOLD HAVE?<br><br>[A] CATTLE, MILK COWS, OR BULLS?<br>[B] HORSES, DONKEYS, OR MULES?<br>[C] GOATS?<br>[D] SHEEP?<br>[E] PIGS?<br>[F] CHICKENS?<br>[G] GUINEA FOWL?<br>[H] DUCKS?<br>[I] OTHER POULTRY?<br>[J] OTHER-1?<br>[K] OTHER-2?<br><br>*If none, record '00'.*<br>*If 95 or more, record '95'.*<br>*If unknown, record '98'.* | How many?<br>Cattle, milk cows, or bulls ... ___ ___<br>Horses, donkeys, or mules ... ___ ___<br>Goats ... ___ ___<br>Sheep ... ___ ___<br>Pigs ... ___ ___<br>Chickens ... ___ ___<br>Guinea fowl ... ___ ___<br>Ducks ... ___ ___<br>Other poultry *(Specify)*<br>____________________.. ___ ___<br>Other-1 *(Specify)*<br>____________________.. ___ ___<br>Other-2 *(Specify)*<br>____________________.. ___ ___ | |
| HC15. DOES ANY MEMBER OF THIS HOUSEHOLD HAVE A BANK ACCOUNT? | Yes ... 1<br>No ... 2 | |
| HC16. HOW MANY HOUSEHOLD MEMBERS ARE REGISTERED WITH THE NATIONAL HEALTH INSURANCE SCHEME? | Number of persons ... __ __<br><br>DK ... 98 | |

| | | |
|---|---|---|
| HH18. *Record the time.* | Hours and minutes __ __ : __ __ | |

HH19. *Go back to the Birth Registration module and fill in key identification information in a new* INDIVIDUAL BIRTH REGISTRATION QUESTIONNAIRE *for every under-5 that has either a line no. entered in BR7 or in BR8.*

*For each questionnaire enter the person's line number in BP4, their name (from HL2) in BP3 and the information for the related under-5, from BR4 and BR5, into BH1.*

HH20. *You now should have both the* INDIVIDUAL BIRTH REGISTRATION QUESTIONNAIRES *and the* WOMEN'S QUESTIONNAIRES, *each of which should identify the person who is to be interviewed.*

*(If you have not yet filled in key identification information for the eligible persons for the* WOMEN'S QUESTIONNAIRE, *do so now.)*

*Do you have any such questionnaires?*

☐ *Yes ⇨ Go to HH21.*

☐ *No ⇨ Complete the front panel of the* HOUSEHOLD QUESTIONNAIRE *to ensure that all interviews for the household have been completed. Thank respondents for their cooperation.*

HH21. *In the table below, fill in the line nos. and names of persons who have to be interviewed with the* WOMEN'S QUESTIONNAIRE *and/or the* INDIVIDUAL BIRTH REGISTRATION QUESTIONNAIRE.

*Use this table to ensure that all such questionnaires are completed.*

*If one of the persons in the table is the current respondent for the Household Questionnaire, you should consider interviewing them first.*

| *Line no.* | *Person name* | *WQ* Check box if Q exists | *IBRQ* Insert number of Qs that exist | *Are the Qs for this person complete?* |
|---|---|---|---|---|
| __ __ | | | | Yes No |
| __ __ | | | | Yes No |
| __ __ | | | | Yes No |
| __ __ | | | | Yes No |
| __ __ | | | | Yes No |
| __ __ | | | | Yes No |
| __ __ | | | | Yes No |
| __ __ | | | | Yes No |
| __ __ | | | | Yes No |
| __ __ | | | | Yes No |

*Key: WQ – Women's Questionnaire*
*IBRQ – Individual Birth Registration Questionnaire*
*Q - Questionnaire*

*WHEN YOU HAVE COMPLETED ALL QUESTIONNAIRES FOR THE HOUSEHOLD, ENSURE YOU HAVE ALSO COMPLETED THE FRONT PANEL OF THE HOUSEHOLD QUESTIONNAIRE.*

*THANK ALL RESPONDENTS FOR THEIR COOPERATION.*

## Interviewer’s Observations

## Field Editor’s Observations

## Supervisor’s Observations

# WOMEN'S QUESTIONNAIRE

| WOMAN'S INFORMATION PANEL | WM |
|---|---|
| *This questionnaire is to be administered to all women 15 through 49 years of age (see Household Listing Form, column HL8). A separate questionnaire should be used for each eligible woman.* | |
| WM1A. BDR+ Cluster number: ___ ___ ___<br>WM1B. Community name: ______________________ | WM2. Household number: __ __ __ \| __ __ |
| WM3. Woman's name:<br>Name____________________________________ | WM4. Woman's line number: ..........___ ___ |
| WM5. Interviewer name and number:<br>Name___________________________ ___ ___ | WM6. Day / Month / Year of interview:<br>___ ___ / ___ ___ / ___ ___ ___ ___ |

*Read the women's consent form for the Women's Questionnaire respondent before going further in the questionnaire.*

*Is permission given?*

☐ *Yes, permission is given* ⇨ *Go to WM10 to record the time and then begin the interview.*

☐ *No, permission is not given* ⇨ *Complete WM7. Discuss this result with your supervisor.*

| | |
|---|---|
| WM7. Result of woman's interview | Completed ............01<br>Not at home ............02<br>Refused ............03<br>Partly completed ............04<br>Incapacitated ............05<br><br>Other (*specify*) ____________________ 96 |

| *Interviewer notes* |
|---|
| |

| |
|---|
| WM8. Field edited by (name and number): ________________________________________ ___ ___ |

| | | |
|---|---|---|
| WM10. *Record the time.* | Hours and minutes ..................__ __ : __ __ | |

| WOMAN'S BACKGROUND | | WB |
|---|---|---|
| WB1. IN WHAT MONTH AND YEAR WERE YOU BORN?<br><br>*Insert '98' (for month), only if unable, after probing, to obtain specific month* | Date of birth<br>Month...............................................__ __<br><br>Year .....................................__ __ __ __ | |
| WB2. HOW OLD ARE YOU?<br><br>*Probe:* HOW OLD WERE YOU AT YOUR LAST BIRTHDAY?<br><br>*Compare and correct WB1 and/or WB2 if inconsistent* | Age (in completed years) ....................__ __ | |
| WB3. HAVE YOU EVER ATTENDED SCHOOL OR PRESCHOOL? | Yes .............................................................1<br>No...............................................................2 | 2⇨WB6 |
| WB4. WHAT IS THE HIGHEST LEVEL OF SCHOOL YOU ATTENDED? | Preschool ...................................................0<br>Primary.......................................................1<br>Junior High School ...................................2<br>Senior High School.....................................3<br>Higher.........................................................4 | 0⇨WB6 |
| WB5. WHAT IS THE HIGHEST GRADE YOU COMPLETED AT THAT LEVEL?<br><br>*If less than 1 grade, enter '00'* | Grade ..................................................__ __ | |
| WB6. WHAT IS YOUR RELIGION? | Catholic 11<br>Protestant (Anglican, Lutheran, Presbyterian, Methodist, etc.) 12<br>Pentecostal/Charismatic 13<br>Other Christian 14<br>Moslem 21<br>Traditional/Spiritualist 31<br><br>Other (*specify*)________________________ 96<br><br>No religion 41 | |
| WB7. TO WHAT ETHNIC GROUP DO YOU BELONG? | Basari 11<br>Ewe 12<br>Fulani 21<br>Gruma 31<br>Kokomba 32<br>Mole-Dagbani 41<br><br>Other (*specify*)______________________ 96 | |

## CHILD MORTALITY CM

NOW I WOULD LIKE TO ASK ABOUT ALL THE PREGNANCIES YOU HAVE HAD DURING YOUR LIFE. BY THIS I MEAN ALL THE CHILDREN BORN TO YOU, WHETHER THEY WERE BORN ALIVE OR DEAD, WHETHER STILL LIVING OR NOT, AND PREGNANCIES WHICH YOU HAVE HAD THAT DID NOT RESULT IN A LIVE BIRTH.

| | | |
|---|---|---|
| CM1. HAVE YOU EVER GIVEN BIRTH? | Yes ....................1<br>No ....................2 | 2⇨CM8 |
| CM2. DO YOU HAVE ANY SONS OR DAUGHTERS TO WHOM YOU HAVE GIVEN BIRTH WHO ARE NOW LIVING WITH YOU? | Yes ....................1<br>No ....................2 | 2⇨CM4 |
| CM3. HOW MANY SONS LIVE WITH YOU?<br>HOW MANY DAUGHTERS LIVE WITH YOU?<br>*If none, record '00'.* | Sons at home __ __<br>Daughters at home __ __ | |
| CM4. DO YOU HAVE ANY SONS OR DAUGHTERS TO WHOM YOU HAVE GIVEN BIRTH WHO ARE ALIVE BUT DO NOT LIVE WITH YOU? | Yes ....................1<br>No ....................2 | 2⇨CM6 |
| CM5. HOW MANY SONS ARE ALIVE BUT DO NOT LIVE WITH YOU?<br>HOW MANY DAUGHTERS ARE ALIVE BUT DO NOT LIVE WITH YOU?<br>*If none, record '00'.* | Sons elsewhere ....................__ __<br>Daughters elsewhere ....................__ __ | |
| CM6. HAVE YOU EVER GIVEN BIRTH TO A BOY OR GIRL WHO WAS BORN ALIVE BUT LATER DIED?<br>*If "No" probe by asking:*<br>I MEAN, TO A CHILD WHO BREATHED OR CRIED OR SHOWED OTHER SIGNS OF LIFE – EVEN IF HE OR SHE LIVED ONLY A FEW MINUTES? | Yes ....................1<br>No ....................2 | 2⇨CM8 |
| CM7. HOW MANY BOYS HAVE DIED?<br>HOW MANY GIRLS HAVE DIED?<br>*If none, record '00'.* | Boys dead ....................__ __<br>Girls dead ....................__ __ | |
| CM8. PREGNANCIES CAN RESULT IN A CHILD THAT IS BORN DEAD, OFTEN CALLED A STILLBIRTH. HAVE YOU EVER HAD A STILLBIRTH? | Yes 1 ⇨ *Ask* HOW MANY STILLBIRTHS? \|__\|__\|<br>No.......... 2 | |
| CM9. WOMEN SOMETIMES HAVE PREGNANCIES THAT DO NOT RESULT IN A LIVE BIRTH AND END BEFORE FULL-TERM, SUCH AS A MISCARRIAGE OR ABORTION. EXCLUDING STILLBIRTHS, HAVE YOU EVER HAD A MISCARRIAGE OR ABORTION? | Yes 1 ⇨ *Ask* IN TOTAL, HOW MANY ABORTIONS AND MISCARRIAGES? \|__\|__\|<br>No......... 2 | |
| CM10. *Sum answers to CM3, CM5 and CM7*<br>*If none, record '00'.* | Sum (live births) ....................__ __ | |
| CM11. *Sum answers to CM8 and CM9*<br>*If none, record '00'.* | Sum (other pregnancies)....................__ __ | |

CM12. JUST TO MAKE SURE I HAVE THIS RIGHT, YOU HAVE HAD IN TOTAL (*total number in CM10*) LIVE BIRTHS DURING YOUR LIFE. IS THIS CORRECT?

☐ Yes ☐ No ⇨ *Probe and correct CM3 toCM7and CM10 as necessary*

CM13. AND YOU HAVE HAD IN TOTAL (*total number in CM11*) STILLBIRTHS, MISCARRIAGES AND ABORTIONS DURING YOUR LIFE. IS THIS CORRECT?

☐ Yes ☐ No ⇨ *Probe and correct CM8, CM9 and CM11 as necessary*

CM14. *Check total of CM10 and CM11*

☐ One or more pregnancies ⇨ *Go to PH1 (in next module)* ☐ No pregnancies ⇨ *Go to PH18*

## PREGNANCY HISTORY PH

NOW I WOULD LIKE TO RECORD ALL YOUR PREGNANCIES, WHETHER BORN ALIVE, BORN DEAD, OR LOST BEFORE FULL TERM. PLEASE START WITH THE FIRST PREGNANCY YOU HAD.

*Record all pregnancies. Record twins and triplets on separate lines.*

| PH1 | PH2 | PH3 | PH4 | PH5 | PH6 | PH7 | PH8 |
|---|---|---|---|---|---|---|---|
| Row No | THINK BACK TO THE TIME OF YOUR (*first/next*) PREGNANCY. WAS THAT A SINGLE OR MULTIPLE PREGNANCY? | WAS THE BABY BORN ALIVE, BORN DEAD OR LOST BEFORE FULL TERM? | DID THAT BABY CRY, MOVE OR BREATHE WHEN IT WAS BORN? | WHAT NAME WAS GIVEN TO THE CHILD? | IS (*name*) A BOY OR A GIRL? | IN WHAT MONTH AND YEAR WAS (*name*) BORN? *Insert '98' (for month), only if unable, after probing, to obtain specific month* | IS (*name*) STILL ALIVE? |
| 01 | Sing .........1<br>Mult..........2 | Born alive. ....................1 ⇨*PH5*<br>Born dead ....................2<br>Lost before full term .....3 ⇨*PH 13* | Yes..........1<br>No............2 ⇨*PH 13* | .____________<br>____________<br>(Name) | Boy........1<br>Girl.........2 | Month ☐☐<br>Year ☐☐☐☐ | Yes..........1<br>No ...........2 ⇨*PH 12* |
| 02 | Sing .........1<br>Mult..........2 | Born alive. ....................1 ⇨*PH5*<br>Born dead ....................2<br>Lost before full term .....3 ⇨*PH 13* | Yes..........1<br>No............2 ⇨*PH 13* | .____________<br>____________<br>(Name) | Boy........1<br>Girl.........2 | Month ☐☐<br>Year ☐☐☐☐ | Yes..........1<br>No ...........2 ⇨*PH 12* |
| 03 | Sing .........1<br>Mult..........2 | Born alive. ....................1 ⇨*PH5*<br>Born dead ....................2<br>Lost before full term .....3 ⇨*PH 13* | Yes..........1<br>No............2 ⇨*PH 13* | .____________<br>____________<br>(Name) | Boy........1<br>Girl.........2 | Month ☐☐<br>Year ☐☐☐☐ | Yes..........1<br>No ...........2 ⇨*PH 12* |
| 04 | Sing .........1<br>Mult..........2 | Born alive. ....................1 ⇨*PH5*<br>Born dead ....................2<br>Lost before full term .....3 ⇨*PH 13* | Yes..........1<br>No............2 ⇨*PH 13* | .____________<br>____________<br>(Name) | Boy........1<br>Girl.........2 | Month ☐☐<br>Year ☐☐☐☐ | Yes..........1<br>No ...........2 ⇨*PH 12* |
| 05 | Sing .........1<br>Mult..........2 | Born alive. ....................1 ⇨*PH5*<br>Born dead ....................2<br>Lost before full term .....3 ⇨*PH 13* | Yes..........1<br>No............2 ⇨*PH 13* | .____________<br>____________<br>(Name) | Boy........1<br>Girl.........2 | Month ☐☐<br>Year ☐☐☐☐ | Yes..........1<br>No ...........2 ⇨*PH 12* |
| 06 | Sing .........1<br>Mult..........2 | Born alive. ....................1 ⇨*PH5*<br>Born dead ....................2<br>Lost before full term .....3 ⇨*PH 13* | Yes..........1<br>No............2 ⇨*PH 13* | .____________<br>____________<br>(Name) | Boy........1<br>Girl.........2 | Month ☐☐<br>Year ☐☐☐☐ | Yes..........1<br>No ...........2 ⇨*PH 12* |
| 07 | Sing .........1<br>Mult..........2 | Born alive. ....................1 ⇨*PH5*<br>Born dead ....................2<br>Lost before full term .....3 ⇨*PH 13* | Yes..........1<br>No............2 ⇨*PH 13* | .____________<br>____________<br>(Name) | Boy........1<br>Girl.........2 | Month ☐☐<br>Year ☐☐☐☐ | Yes..........1<br>No ...........2 ⇨*PH 12* |
| 08 | Sing .........1<br>Mult..........2 | Born alive. ....................1 ⇨*PH5*<br>Born dead ....................2<br>Lost before full term .....3 ⇨*PH 13* | Yes..........1<br>No............2 ⇨*PH 13* | .____________<br>____________<br>(Name) | Boy........1<br>Girl.........2 | Month ☐☐<br>Year ☐☐☐☐ | Yes..........1<br>No ...........2 ⇨*PH 12* |

| *If born alive and still living* | | | *If born alive, but now dead* | *If born dead or lost before birth* | | | |
|---|---|---|---|---|---|---|---|
| PH9 | PH10 | PH11 | PH12 | PH13 | PH14 | PH15 | PH16 |
| HOW OLD WAS (*name*) AT HIS/HER LAST BIRTHDAY?<br><br>*Record age in completed years.* | IS (*name*) LIVING WITH YOU? | *Record household line number of child.*<br><br>*(Record '00' if child not listed in household)* | HOW OLD WAS (*name*) WHEN SHE/HE DIED?<br>*If '1 year' probe:*<br>HOW MANY MONTHS OLD WAS (*name*)?<br><br>*Record days if less than 1 month, months if less than 2 years, or years.* | IN WHAT MONTH AND YEAR DID THIS PREGNANCY END?<br><br>*Insert '98' (for month), only if unable, after probing, to obtain specific month* | HOW MANY MONTHS DID THE PREGNANCY LAST?<br><br>*Record in completed months.* | DID YOU OR SOMEONE ELSE DO SOMETHING TO END THAT PREGNANCY?<br><br>*Ask only if PH3 has code '3' circled* | WERE THERE ANY OTHER PREGNANCIES BETWEEN THE PREVIOUS PREGNANCY AND THIS ONE? |
| 01<br>Age in years | Yes..........1<br>No ...........2<br>⇨ *(Next pregnancy)* | Line number<br>⇨ *(Next pregnancy)* | Days .......... 1<br>Months ..... 2<br>Years ......... 3<br>⇨ *(Next pregnancy)* | Month<br>Year | Months | Yes..........1<br>No ...........2 | |
| 02<br>Age in years | Yes..........1<br>No ...........2<br>⇨ *PH16* | Line number<br>⇨ *PH16* | Days .......... 1<br>Months ..... 2<br>Years ........ 3<br>⇨ *PH16* | Month<br>Year | Months | Yes..........1<br>No ...........2 | Yes ......... 1<br>No........... 2 |
| 03<br>Age in years | Yes..........1<br>No ...........2<br>⇨ *PH16* | Line number<br>⇨ *PH16* | Days .......... 1<br>Months ..... 2<br>Years ........ 3<br>⇨ *PH16* | Month<br>Year | Months | Yes..........1<br>No ...........2 | Yes ......... 1<br>No........... 2 |
| 04<br>Age in years | Yes..........1<br>No ...........2<br>⇨ *PH16* | Line number<br>⇨ *PH16* | Days .......... 1<br>Months ..... 2<br>Years ........ 3<br>⇨ *PH16* | Month<br>Year | Months | Yes..........1<br>No ...........2 | Yes ......... 1<br>No........... 2 |
| 05<br>Age in years | Yes..........1<br>No ...........2<br>⇨ *PH16* | Line number<br>⇨ *PH16* | Days .......... 1<br>Months ..... 2<br>Years ........ 3<br>⇨ *PH16* | Month<br>Year | Months | Yes..........1<br>No ...........2 | Yes ......... 1<br>No........... 2 |
| 06<br>Age in years | Yes..........1<br>No ...........2<br>⇨ *PH16* | Line number<br>⇨ *PH16* | Days .......... 1<br>Months ..... 2<br>Years ........ 3<br>⇨ *PH16* | Month<br>Year | Months | Yes..........1<br>No ...........2 | Yes ......... 1<br>No........... 2 |
| 07<br>Age in years | Yes..........1<br>No ...........2<br>⇨ *PH16* | Line number<br>⇨ *PH16* | Days .......... 1<br>Months ..... 2<br>Years ........ 3<br>⇨ *PH16* | Month<br>Year | Months | Yes..........1<br>No ...........2 | Yes ......... 1<br>No........... 2 |
| 08<br>Age in years | Yes..........1<br>No ...........2<br>⇨ *PH16* | Line number<br>⇨ *PH16* | Days .......... 1<br>Months ..... 2<br>Years ........ 3<br>⇨ *PH16* | Month<br>Year | Months | Yes..........1<br>No ...........2 | Yes ......... 1<br>No........... 2 |

## PREGNANCY HISTORY (continued) PH

*This is a continuation from row no. 8 on the last page.*

*Record all pregnancies. Record twins and triplets on separate lines.*

| PH1 | PH2 | PH3 | PH4 | PH5 | PH6 | PH7 | PH8 |
|---|---|---|---|---|---|---|---|
| Row No | THINK BACK TO THE TIME OF YOUR (*first/next*) PREGNANCY. WAS THAT A SINGLE OR MULTIPLE PREGNANCY? | WAS THE BABY BORN ALIVE, BORN DEAD OR LOST BEFORE FULL TERM? | DID THAT BABY CRY, MOVE OR BREATHE WHEN IT WAS BORN? | WHAT NAME WAS GIVEN TO THE CHILD? | IS (*name*) A BOY OR A GIRL? | IN WHAT MONTH AND YEAR WAS (*name*) BORN? *Insert '98' (for month), only if unable, after probing, to obtain specific month* | IS (*name*) STILL ALIVE? |
| 09 | Sing .........1<br>Mult..........2 | Born alive. ....................1 ⇨*PH5*<br>Born dead ....................2<br>Lost before full term .....3 ⇨*PH 13* | Yes..........1<br>No............2 ⇨*PH 13* | .____________<br>____________<br>(Name) | Boy........1<br>Girl.........2 | Month ☐☐<br>Year ☐☐☐☐ | Yes..........1<br>No ...........2 ⇨*PH 12* |
| 10 | Sing .........1<br>Mult..........2 | Born alive. ....................1 ⇨*PH5*<br>Born dead ....................2<br>Lost before full term .....3 ⇨*PH 13* | Yes..........1<br>No............2 ⇨*PH 13* | .____________<br>____________<br>(Name) | Boy........1<br>Girl.........2 | Month ☐☐<br>Year ☐☐☐☐ | Yes..........1<br>No ...........2 ⇨*PH 12* |
| 11 | Sing .........1<br>Mult..........2 | Born alive. ....................1 ⇨*PH5*<br>Born dead ....................2<br>Lost before full term .....3 ⇨*PH 13* | Yes..........1<br>No............2 ⇨*PH 13* | .____________<br>____________<br>(Name) | Boy........1<br>Girl.........2 | Month ☐☐<br>Year ☐☐☐☐ | Yes..........1<br>No ...........2 ⇨*PH 12* |
| 12 | Sing .........1<br>Mult..........2 | Born alive. ....................1 ⇨*PH5*<br>Born dead ....................2<br>Lost before full term .....3 ⇨*PH 13* | Yes..........1<br>No............2 ⇨*PH 13* | .____________<br>____________<br>(Name) | Boy........1<br>Girl.........2 | Month ☐☐<br>Year ☐☐☐☐ | Yes..........1<br>No ...........2 ⇨*PH 12* |
| 13 | Sing .........1<br>Mult..........2 | Born alive. ....................1 ⇨*PH5*<br>Born dead ....................2<br>Lost before full term .....3 ⇨*PH 13* | Yes..........1<br>No............2 ⇨*PH 13* | .____________<br>____________<br>(Name) | Boy........1<br>Girl.........2 | Month ☐☐<br>Year ☐☐☐☐ | Yes..........1<br>No ...........2 ⇨*PH 12* |
| 14 | Sing .........1<br>Mult..........2 | Born alive. ....................1 ⇨*PH5*<br>Born dead ....................2<br>Lost before full term .....3 ⇨*PH 13* | Yes..........1<br>No............2 ⇨*PH 13* | .____________<br>____________<br>(Name) | Boy........1<br>Girl.........2 | Month ☐☐<br>Year ☐☐☐☐ | Yes..........1<br>No ...........2 ⇨*PH 12* |
| 15 | Sing .........1<br>Mult..........2 | Born alive. ....................1 ⇨*PH5*<br>Born dead ....................2<br>Lost before full term .....3 ⇨*PH 13* | Yes..........1<br>No............2 ⇨*PH 13* | .____________<br>____________<br>(Name) | Boy........1<br>Girl.........2 | Month ☐☐<br>Year ☐☐☐☐ | Yes..........1<br>No ...........2 ⇨*PH 12* |
| 16 | Sing .........1<br>Mult..........2 | Born alive. ....................1 ⇨*PH5*<br>Born dead ....................2<br>Lost before full term .....3 ⇨*PH 13* | Yes..........1<br>No............2 ⇨*PH 13* | .____________<br>____________<br>(Name) | Boy........1<br>Girl.........2 | Month ☐☐<br>Year ☐☐☐☐ | Yes..........1<br>No ...........2 ⇨*PH 12* |

| *If born alive and still living* | | | *If born alive, but now dead* | *If born dead or lost before birth* | | | |
|---|---|---|---|---|---|---|---|
| PH9 | PH10 | PH11 | PH12 | PH13 | PH14 | PH15 | PH16 |
| HOW OLD WAS (*name*) AT HIS/HER LAST BIRTHDAY?<br><br>*Record age in completed years.* | IS (*name*) LIVING WITH YOU? | *Record household line number of child.*<br><br>*(Record '00' if child not listed in household)* | HOW OLD WAS (*name*) WHEN SHE/HE DIED?<br>*If '1 year' probe*:<br>HOW MANY MONTHS OLD WAS (*name*)?<br><br>*Record days if less than 1 month, months if less than 2 years, or years.* | IN WHAT MONTH AND YEAR DID THIS PREGNANCY END?<br><br>*Insert '98' (for month), only if unable, after probing, to obtain specific month* | HOW MANY MONTHS DID THE PREGNANCY LAST?<br><br>*Record in completed months.* | DID YOU OR SOMEONE ELSE DO SOMETHING TO END THAT PREGNANCY?<br><br>*Ask only if PH3 has code '3' circled* | WERE THERE ANY OTHER PREGNANCIES BETWEEN THE PREVIOUS PREGNANCY AND THIS ONE? |
| 09<br>Age in years | Yes..........1<br>No ...........2<br>⇨ *PH16* | Line number<br>⇨ *PH16* | Days .......... 1<br>Months ..... 2<br>Years ......... 3<br>⇨ *PH16* | Month<br>Year | Months | Yes..........1<br>No ...........2 | Yes ......... 1<br>No........... 2 |
| 10<br>Age in years | Yes..........1<br>No ...........2<br>⇨ *PH16* | Line number<br>⇨ *PH16* | Days .......... 1<br>Months ..... 2<br>Years ........ 3<br>⇨ *PH16* | Month<br>Year | Months | Yes..........1<br>No ...........2 | Yes ......... 1<br>No........... 2 |
| 11<br>Age in years | Yes..........1<br>No ...........2<br>⇨ *PH16* | Line number<br>⇨ *PH16* | Days .......... 1<br>Months ..... 2<br>Years ........ 3<br>⇨ *PH16* | Month<br>Year | Months | Yes..........1<br>No ...........2 | Yes ......... 1<br>No........... 2 |
| 12<br>Age in years | Yes..........1<br>No ...........2<br>⇨ *PH16* | Line number<br>⇨ *PH16* | Days .......... 1<br>Months ..... 2<br>Years ........ 3<br>⇨ *PH16* | Month<br>Year | Months | Yes..........1<br>No ...........2 | Yes ......... 1<br>No........... 2 |
| 13<br>Age in years | Yes..........1<br>No ...........2<br>⇨ *PH16* | Line number<br>⇨ *PH16* | Days .......... 1<br>Months ..... 2<br>Years ........ 3<br>⇨ *PH16* | Month<br>Year | Months | Yes..........1<br>No ...........2 | Yes ......... 1<br>No........... 2 |
| 14<br>Age in years | Yes..........1<br>No ...........2<br>⇨ *PH16* | Line number<br>⇨ *PH16* | Days .......... 1<br>Months ..... 2<br>Years ........ 3<br>⇨ *PH16* | Month<br>Year | Months | Yes..........1<br>No ...........2 | Yes ......... 1<br>No........... 2 |
| 15<br>Age in years | Yes..........1<br>No ...........2<br>⇨ *PH16* | Line number<br>⇨ *PH16* | Days .......... 1<br>Months ..... 2<br>Years ........ 3<br>⇨ *PH16* | Month<br>Year | Months | Yes..........1<br>No ...........2 | Yes ......... 1<br>No........... 2 |
| 16<br>Age in years | Yes..........1<br>No ...........2<br>⇨ *PH16* | Line number<br>⇨ *PH16* | Days .......... 1<br>Months ..... 2<br>Years ........ 3<br>⇨ *PH16* | Month<br>Year | Months | Yes..........1<br>No ...........2 | Yes ......... 1<br>No........... 2 |

| | | |
|---|---|---|
| PH17. HAVE YOU HAD ANY PREGNANCY SINCE THE LAST PREGNANCY MENTIONED?<br>*Exclude current pregnancy* | Yes ........................................ 1<br>No........................................... 2 | 1⇨PH1 |
| PH18. ARE YOU PREGNANT NOW? | Yes ........................................ 1<br>No........................................... 2<br>DK ........................................ 8 | <br>2⇨PH20<br>8⇨PH20 |
| PH19. HOW MANY MONTHS PREGNANT ARE YOU? | ☐☐ Months | |

PH20. *Count number of live births by counting rows with PH6 code circled for boy or girl.*

*Enter total* → |__|__|

*Is this number the same as for CM10*

☐ Yes ☐ No ⇨ *Probe and reconcile as necessary*

PH21. *Count number of other pregnancies by counting rows without PH6 code circled for boy or girl.*

*Enter total* → |__|__|

*Is this number the same as for CM11*

☐ Yes ☐ No ⇨ *Probe and reconcile as necessary*

PH22. *Review the following items in the Pregnancy History module and check each box when completed*

- *For each pregnancy, year is recorded in PH7 or PH13* ☐
- *For each living child: current age is recorded in PH9* ☐
- *For each dead child: Age of death is recorded in PH12* ☐
- *For age at death 12 months or 1 year, probe for exact number of months* ☐

PH23. *Check Pregnancy History module. Did last live birth occur within the last two years, that is, since (day and month of interview) in* ***2011?***

☐ No live birth in last 2 years. ⇨ *Go to PH24 (immediately after end of Maternal Health module).*

☐ One or more live births in last 2 years. ⇨ *Ask for the name of the child*

Name of child________________________________

*If child has died, take special care when referring to this child by name in the next module.*

*Continue with the next module.*

## MATERNAL HEALTH — MH

*This module is to be administered to all women with a live birth in the 2 years preceding date of interview.*

*Check PH21 and record name of last-born child here:* ______________________________ *Use this name below*

| | | |
|---|---|---|
| MH1. DID YOU SEE ANYONE FOR ANTENATAL CARE DURING YOUR PREGNANCY WITH (*name*)? | Yes ....... 1<br>No ....... 2 | 2⇨MH4 |
| MH2. WHOM DID YOU SEE?<br><br>*Probe:*<br>ANYONE ELSE?<br><br>*Probe for the type of person seen and circle all answers given.* | Health professional:<br>Doctor ....... A<br>Nurse / Midwife ....... B<br>Other person<br>Traditional birth attendant ....... F<br>Community health worker ....... G<br><br>Other (*specify*) ______________ X | |
| MH3. HOW MANY TIMES DID YOU RECEIVE ANTENATAL CARE DURING THIS PREGNANCY? | Number of times ....... __ __<br><br>DK ....... 98 | |
| MH4. WHO ASSISTED WITH THE DELIVERY OF (*name*)?<br><br>*Probe:*<br>ANYONE ELSE?<br><br>*Probe for the type of person assisting and circle all answers given.*<br><br>*If respondent says no one assisted, probe to determine whether any adults were present at the delivery.* | Health professional:<br>Doctor ....... A<br>Nurse / Midwife ....... B<br>Other person<br>Traditional birth attendant ....... F<br>Community health worker ....... G<br>Relative / Friend ....... H<br><br>Other (*specify*) ______________ X<br>No one ....... Y | |
| MH5. WHERE DID YOU GIVE BIRTH TO (*name*)?<br><br>*Probe to identify the type of source.*<br><br>*If unable to determine whether public or private, write the name of the place.*<br><br>______________________<br>*(Name of place)* | Home<br>Your home ....... 11<br>Other home ....... 12<br>Public sector<br>Govt. hospital ....... 21<br>Govt. clinic / health centre ....... 22<br>Govt. health post ....... 23<br>Other public (*specify*) __________ 26<br>Private Medical Sector<br>Private hospital ....... 31<br>Private clinic ....... 32<br>Private maternity home ....... 33<br>Other private<br>medical (*specify*) __________ 36<br><br>Other (*specify*) ______________ 96 | |

PH24. *Check Pregnancy History module to identify if there has been a death of a child under 5 years of age in the last five years*

☐ Yes ⇨ *Go to next module.*

☐ No ⇨ *Go to WM11*

| **REGISTRATION OF A DEATH** | | **RD** |
|---|---|---|
| WHEN I ASKED YOU EARLIER ABOUT YOUR PREVIOUS BIRTHS, YOU TOLD ME THAT ONE OF YOUR CHILDREN, WHO WAS UNDER 5 YEARS OF AGE, DIED IN THE LAST FIVE YEARS. | | |
| RD1. WHERE DID THIS CHILD'S DEATH OCCUR: AT HOME, IN A HEALTH FACILITY, OR SOMEWHERE ELSE? | Home .......... 1<br>Health facility .......... 2<br>Other *(specify)* ______________ 6 | |
| RD2. WHERE WAS THE CHILD BURIED: IN A CEMETERY, JUST OUTSIDE YOUR COMPOUND, WITHIN YOUR COMPOUND, OR ELSEWHERE? | Cemetery .......... 1<br>Just outside your compound .......... 2<br>Within your compound .......... 3<br>Other *(specify)* ______________ 6 | |
| RD3. WAS AN AUTHORIZATION FOR BURIAL OBTAINED FOR THIS CHILD? | Yes .......... 1<br>No .......... 2<br>DK .......... 8 | <br>2⇨RD6<br>8⇨RD6 |
| RD4. FROM WHOM WAS THIS AUTHORIZATION OBTAINED? | Chief of community .......... 1<br>Imam .......... 2<br>Priest .......... 3<br>CBV .......... 4<br>Nurse or Midwife .......... 5<br>Other *(specify)* ______________ 6 | |
| RD5. WAS THE DEATH OF THIS CHILD REPORTED TO ANYONE ELSE OF IMPORTANCE IN THE COMMUNITY, SUCH AS THE CHIEF OR THE IMAM? | Yes .......... 1<br>No .......... 2<br>DK .......... 8 | 1⇨RD7<br>2⇨WM11<br>8⇨WM11 |
| RD6. WAS THE DEATH REPORTED TO SOMEONE OF IMPORTANCE IN THE COMMUNITY, SUCH AS THE CHIEF, THE IMAM OR THE PRIEST? | Yes .......... 1<br>No .......... 2 | <br>2⇨WM11 |
| RD7. WHO WAS THIS PERSON?<br>*Probe:*<br>ANYONE ELSE?<br>*Probe for the type of person and circle all answers given.* | Chief of community .......... A<br>Imam .......... B<br>Priest .......... C<br>CBV .......... D<br>Nurse or Midwife .......... E<br>Other *(specify)* ______________ X | |

| | | |
|---|---|---|
| WM11. *Record the time.* | Hours and minutes .......... __ __ : __ __ | |

WM12. *Go to HH21 at the end of the Household Questionnaire and check if there are INDIVIDUAL BIRTH REGISTRATION QUESTIONNAIRES that the present respondent should be interviewed for.*

*Are there any such questionnaires?*

☐ Yes ⇨ *Start the interview using the appropriate INDIVIDUAL BIRTH REGISTRATION QUESTIONNAIRE.*

☐ No ⇨ *Thank the respondent for her cooperation. Go to HH21 to determine if there are any other interviews to be completed for this household.*

## Interviewer's Observations

## Field Editor's Observations

## Supervisor's Observations

# INDIVIDUAL BIRTH REGISTRATION QUESTIONNAIRE

| **BIRTH REGISTRATION INFORMATION PANEL** | **BP** |
|---|---|
| *This questionnaire is to be administered to each person who is identified in the Household Questionnaire as eligible. See HH21 in the Household Questionnaire for the eligible persons in the present household. The information for BP3, BP4 and BH1 should have already been entered.* | |
| BP1A. BDR+ Cluster number: ___ ___ ___<br>BP1B. Community name: ____________________ | BP2. Household number: __ __ __ \| __ __ |
| BP3. Person's name:<br>Name__________________________________ | BP4. Person's line number: ............ ___ ___ |
| BP5. Interviewer name and number:<br>Name_________________________ ___ ___ | BP6. Day / Month / Year of interview:<br>___ ___ / ___ ___ / ___ ___ ___ ___ |

| | |
|---|---|
| BP7. Result of person's interview | Completed ............................................................01<br>Not at home ..........................................................02<br>Refused ................................................................03<br>Partly completed ...................................................04<br>Incapacitated ........................................................05<br><br>Other (*specify*) ___________________________ 96 |

| *Interviewer notes* |
|---|
| |

| |
|---|
| BP8. Field edited by (name and number): _____________________________________ ___ ___ |

| | | |
|---|---|---|
| BP9. *Record the time.* | Hours and minutes __ __ : __ __ | |

| BIRTH REGISTRATION HISTORY OF UNDER-5 | | BH |
|---|---|---|
| BH1. Child's name ________________________ Line no __ __ Age __ __ Sex ______ | | |
| BH2. I UNDERSTAND THAT YOU REGISTERED THE BIRTH OF (*name*). IS THIS CORRECT? | Yes ....... 1<br>No ....... 2 | 1 ⇨ BH5<br>2 ⇨ BH3 |
| BH3. WHO DID REGISTER THE BIRTH OF (*name*)?<br>*If line no. of another person given on right. Correct the BR module and HH21 in the Household Questionnaire. Interview this new person with a new MLS4 form..* | Line no. of person ....... __ __<br>Was never registered ....... 97 | ⇨ BP10<br>97 ⇨ BH4 |
| BH4. WHY WAS (*name*)'S BIRTH NOT REGISTERED? | Costs too much ....... 1<br>Must travel too far ....... 2<br>Did not know the birth should be registered ....... 3<br>Did not know where to register ....... 4<br>Did not want to pay fine ....... 5<br>Other (*specify*) ______________ 6<br>DK ....... 8 | ⇨ BP10 |
| BH5. WHAT IS YOUR RELATIONSHIP TO (*name*)? | Mother ....... 1<br>Father ....... 2<br>Other (*Specify*): ______________ 6 | |
| BH6. DOES (*name*) HAVE A BIRTH CERTIFICATE? MAY I SEE IT?<br>*Check birth certificate to see if it is the one issued by the Births and Deaths Registry* | Yes, seen ....... 1<br>Yes, not seen ....... 2<br>No ....... 3<br>DK ....... 8 | 1 ⇨BH7<br>2 ⇨BH8<br>3⇨BH10<br>8⇨BH10 |
| BH7. WHAT WAS THE DATE OF BIRTH ON THE BIRTH CERTIFICATE?<br>. | Day [ ][ ] Month [ ][ ] Year [ ][ ][ ][ ] | ⇨BH9 |
| BH8. WHAT WAS THE DATE OF BIRTH ON THE BIRTH CERTIFICATE?<br>*Insert '98' for month only if unable, after probing, to obtain the specific month.* | Calendar → Month [ ][ ] Year [ ][ ][ ][ ] | |
| BH9. HOW MUCH TIME DID IT TAKE FROM WHEN YOU REPORTED THE BIRTH TO WHEN YOU RECEIVED THE CERTIFICATE?<br>*Weeks if less than 1 month, months otherwise.* | Weeks (01, 02, 03, 04) [ ][ ]<br>Months (From 01 to 12) [ ][ ] | ⇨BH11 |
| BH10. WHAT WAS THE DATE WHEN YOU REPORTED THE BIRTH OF (*name*)? | Calendar → Month [ ][ ] Year [ ][ ][ ][ ] | |
| BH11. WHAT WAS (*name*)'S AGE AT THE TIME OF REPORTING THE BIRTH?<br>*Enter age in months; insert '00' if less than 1 month* | Age → [ ][ ] In months | |

| | | |
|---|---|---|
| BH12. How much did it cost to register (*name*)'s birth? | 1 Cedi ....1<br>2 Cedis ....2<br>3 Cedis ....3<br>4 Cedis ....4<br>5 Cedis ....5<br>More than 5 Cedis ....6<br>Free ....7<br>DK....8 | |
| BH13. Why did you want to register (*name*)'s birth? Was it …<br>[A] For school?<br>[B] For an official government document?<br>[C] For National Health Insurance?<br>[D] For another reason? | Yes No<br>School ....1 2<br>Govt. document ....1 2<br>NHI ....1 2<br>Other (*specify*)______ | |
| BH14. Where did you register (*name*)'s birth? | BDR Office ....1<br>In this Community....2<br>Other (*specify*)______ 6 | |
| BH15. Who did you register (*name*)'s birth with: the BDR officer, the BDR officer's assistant, the Community Based Volunteer (CBV), or someone else? | BDR Officer ....1<br>BDR Officer's Assistant....2<br>CBV ....3<br>Other (*specify*)______ 6 | |
| BH16. How satisfied were you with the birth registration process? Were you satisfied, partly satisfied or not satisfied at all? | Satisfied....1<br>Partly satisfied ....2<br>Not satisfied....3 | |
| BH17. If you were to register another birth, would you want to go to the same person or someone different? | Same ....1<br>Different....2 | 2⇨BH19 |
| BH18. Why would you want to go to the same person again? Was it because …<br>[A]. Person is nearby/Not far?<br>[B]. Person was nice / friendly?<br>[C]. Short time to wait for certificate?<br>[D]. Of another reason? | Yes No<br>Near ....1 2<br>Nice ....1 2<br>Short time ....1 2<br>Other (*specify*)______ | ➔ BH20 |
| BH19. Why would you want to go to a different person? Was it because …<br>[A]. Person too far away ?<br>[B]. Person was not nice / not friendly?<br>[C]. Long wait before getting certificate?<br>[D]. Of another reason | Yes No<br>Too far ....1 2<br>Not nice ....1 2<br>Long wait ....1 2<br>Other (*specify*)______ | |
| BH20. Do you know birth registration is free if you register a child before they are a certain age? | Yes ....1<br>No ....2<br>DK ....8 | 2⇨BP10<br>8⇨BP10 |
| BH21. Up to what age, in months, is birth registration free? | Age → [ ][ ] In months | |

| | | |
|---|---|---|
| BP10. *Record the time.* | Hours and minutes __ __ : __ __ | |
| BP11. *Thank the respondent and go back toHH21 in the Household Questionnaire to determine what other interviews need to be completed for the present household* | | |

## Interviewer’s Observations

## Field Editor’s Observations

## Supervisor’s Observations
