# Supplementary material for: Using community-based reporting of vital events to monitor child mortality: Lessons from rural Ghana
Source: PLoS One. 2018 Jan 30;13(1):e0192034. doi: 10.1371/journal.pone.0192034 (PMC5790256; doi:10.1371/journal.pone.0192034)

Supporting Information File 4: Assessment of the quality of FPH data

We investigated the quality of FPH data in several ways. First, we assessed the distribution of the population by age and gender listed during the census. It is represented in Figure A4.1. The population pyramid shows large recent birth cohorts (e.g., age groups 0-4 and 5-9 years old), consistent with the high fertility rates observed throughout the Northern Region of Ghana. There are also noticeable irregularities, particularly regarding the age distribution among women. Women aged 15-19 years appear under-represented, as they constitute less than 6% of the household population, i.e., a much smaller share than immediately neighboring age groups (12% for 10-14 years old, and >7% for 20-24 years old). This may be due to several factors, such as migration for schooling purposes (boarding schools). It may, however, also result from inaccurate age reporting, with a number of women aged 15-19 years "displaced" into neighboring age groups. Similarly, we found that women aged 45-49 years old were largely under-represented in the population, whereas women aged 50-54 were over-represented. This is likely due to significant age heaping during the end-of project census (see below). These irregularities may result in the under-reporting of births and child deaths during the FPH, compared with the CBV data.

<u>Figure A4.1</u>. Population distribution by age and gender.

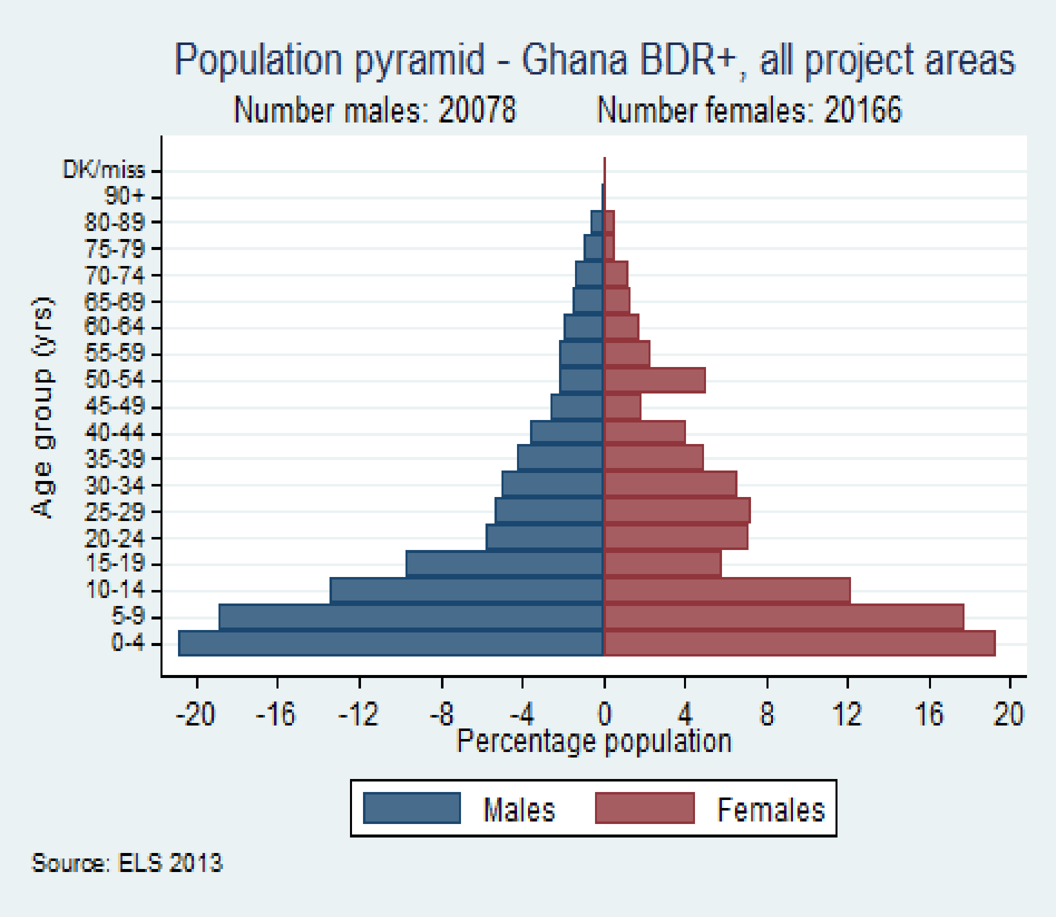

Second, we also measured heaping in the reporting of mothers' ages during the end-of-project census. This is commonly used as a diagnostic of the quality of demographic data collected during interviews. Figure A4.2 shows significant heaping in ages ending in -0 or -5 during the end-of-project census, across the entire 15-49 years age span. Heaping was most pronounced in Zabzugu, where four times more respondents than expected reported being 30 years old, for example (Figure A4.2).

Figure A4.2. Reported maternal age in end-of-project census data, by district.

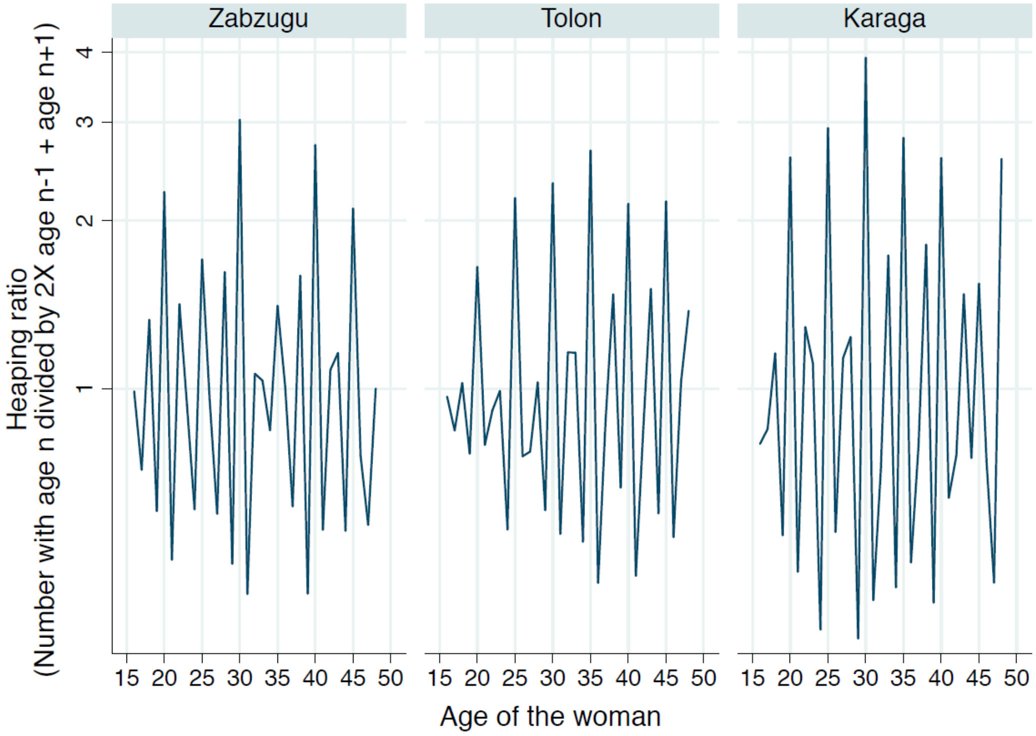

Third, we then measured changes in the number of births recorded by year, to investigate possible displacements of births outside of the study period. These results are presented in Figure A4.3. They indicate variation in the number of births reported per year. For example, in Tolon, this analysis indicates that some births may have been displaced to 2007, i.e., 6 years prior to the survey. Similarly, in Zabzugu, some births may have been displaced to 2002, i.e., 11 years before the survey.

Figure A4.3. Reported births per year in end-of-project census data, by district.

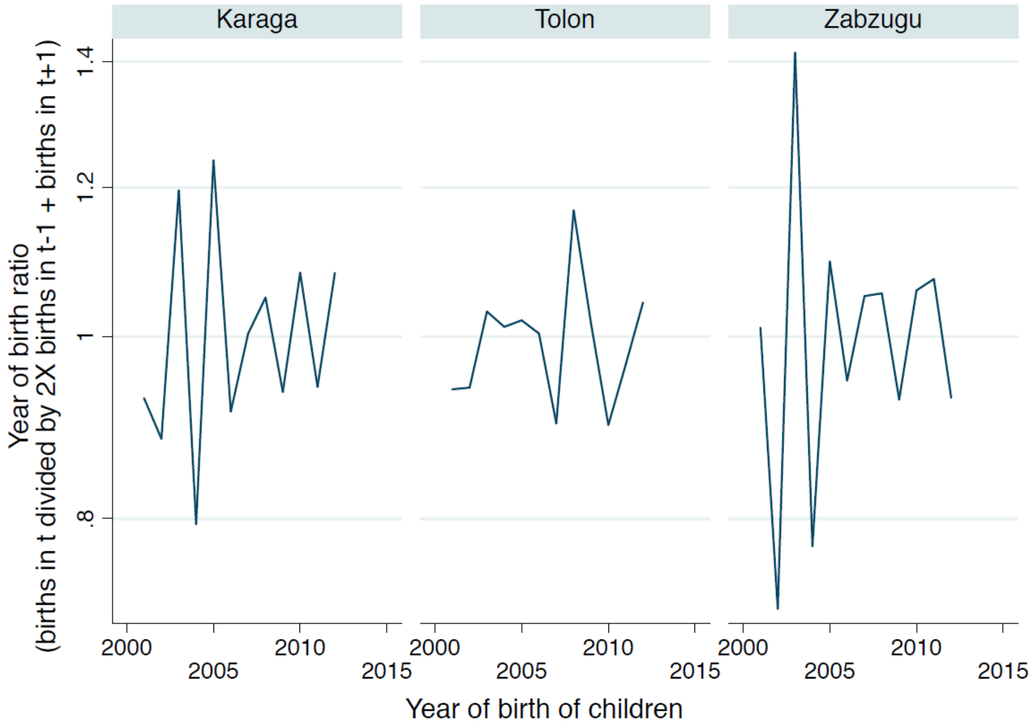

Fourth, we also investigated changes in the reported proportion of pregnancies that ended in a live birth by year. Changes in this proportion may indicate omissions of pregnancies that did not result in a live birth, but it may also point to likely misclassifications of neonatal (and possibly other under-five) deaths as stillbirths. Such misclassifications may be common since the mother may not easily detect some early signs of life. They may also be related to the inclusion of a set of questions that concern only live-born children. For example, interviewers may have an incentive to classify pregnancies as having ended in a stillbirth if this allows skipping a large number of questions related to post-natal care, breastfeeding or vaccinations, as is currently the case in the DHS. We found that the proportion of pregnancies resulting in a live birth declined significantly over time in all districts (Figure A4.4).

<u>Figure A4.4</u>. Proportion of live births among reported pregnancies over time in end-of-project census data, by district.

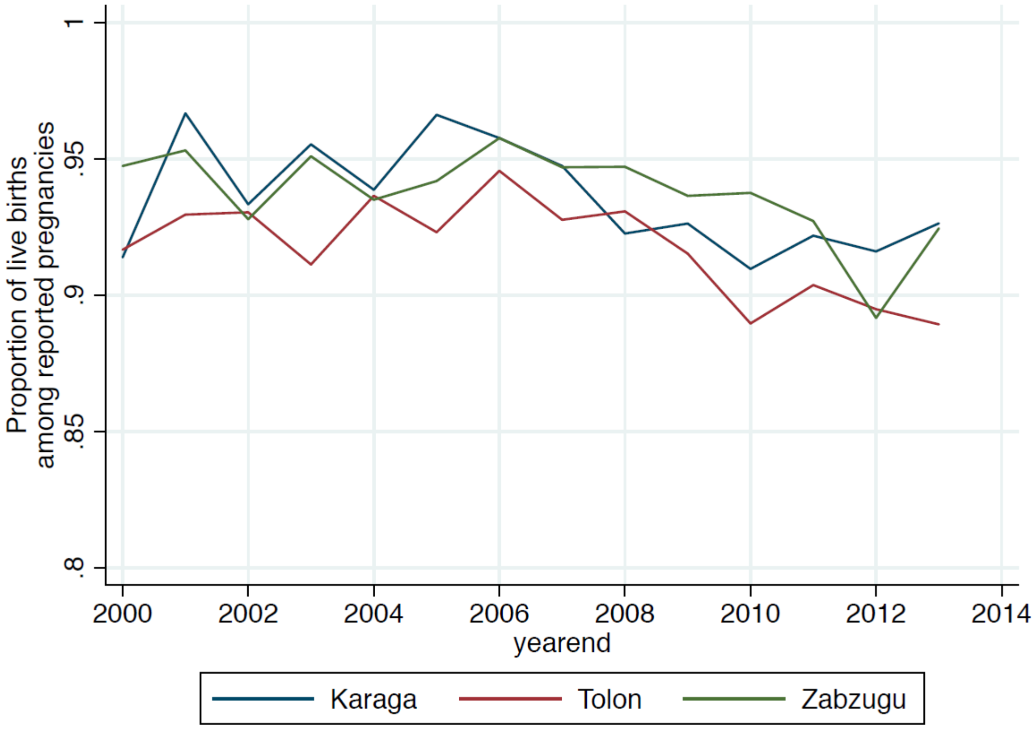

The questionnaire that was used during this project included a) maternal health questions about all live births of a respondent in the past 2 years before the census, and b) questions about the registration of all births and deaths having occurred in the past 5 years (see Webannex 3). In figure A4.5, we thus investigated the distributions of reported births and deaths by year. We calculated the proportions of pregnancies reported to have resulted in a miscarriage, stillbirth, or abortion (“not a live birth”), a neonatal death, or a post-neonatal death, by year. Sharp changes in these distributions across the 2- and 5-year thresholds described above may be due to a series of factors affecting fertility and/or pregnancy outcomes. But they may also indicate the presence of misclassifications and/or displacements in the FPH data due to questionnaire design and interviewer behaviors.

Figure A4.5 shows trends in the reporting of pregnancy outcomes in FPH data. In Karaga, the proportion of reported pregnancies that did not result in a live birth increased sharply, from less than 50% in 2007 to more than 70% in 2008, i.e., at the 5-year threshold requiring the collection of the birth/death registration questions. Similarly in Zabzugu, this proportion increased from 42% in 2010 to more than 70% in 2011, i.e., at the 2-year threshold requiring the collection of the maternal health questions. In Tolon, there was no evidence of a sudden change in the distribution of pregnancy outcomes at either of these thresholds.

These analyses indicate thus that some changes in the reporting of pregnancy outcomes may have been related to questionnaire design and attempts by interviewers to skip questions focused on children under two years old or under five years old. These changes may thus indicative of an increased rate of misclassification for the period immediately preceding the survey, at least in Zabzugu. As a result, the number of neonatal deaths in the period of CBV data collection (2012-2013) may be underestimated in the FPH dataset in Zabzugu.

Figure A4.5. Distribution of pregnancies ending in miscarriage, stillbirth, or infant death in end-of-project census data, by year and district.

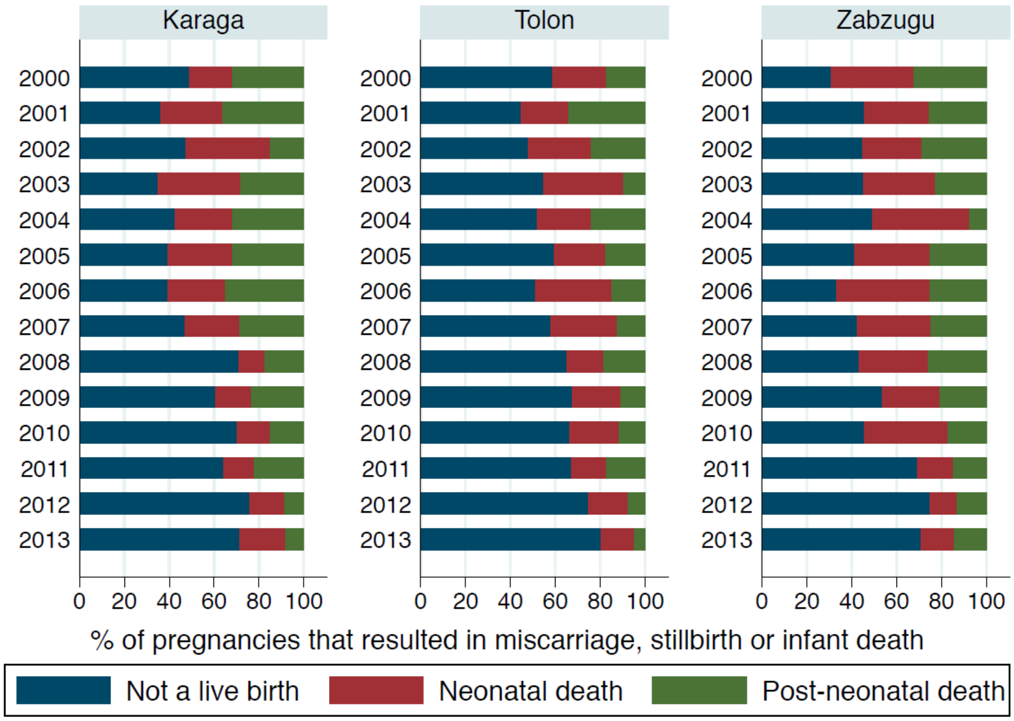

Supplement: S4 File — (PDF) [file pone.0192034.s004.pdf]
